# Glutathione-depleting Liposome Adjuvant for Augmenting the Efficacy of a Glutathione Covalent Inhibitor Oridonin for Acute Myeloid Leukemia Therapy

**DOI:** 10.1186/s12951-024-02574-6

**Published:** 2024-05-30

**Authors:** Yi Liu, Xiaoning Wang, Hui Feng, Xinyan Li, Runyu Yang, Mengyao Zhang, Yue Du, Ruimin Liu, Minna Luo, Zhiyi Li, Bo Liu, Jincheng Wang, Wenjuan Wang, Feifei An, Fan Niu, Pengcheng He

**Affiliations:** 1 Department of Hematology, The First Affiliated Hospital of Xi’an Jiaotong University, No. 277 Yanta West Road, Xi’an, Shaanxi, 710061 China; 2 Department of Urology, The First Affiliated Hospital of Xi’an Jiaotong University, No. 277 Yanta West Road, Xi’an, Shaanxi, 710061 China; 3 School of Public Health, Health Science Center, Xi’an Jiaotong University, No.76 Yanta West Road, Xi’an, Shaanxi, 710061 China

**Keywords:** Oridonin, Peptide-based drug delivery, Glutathione inhibitor, ROS, AML therapeutics

## Abstract

**Background:**

Discrepancies in the utilization of reactive oxygen species (ROS) between cancer cells and their normal counterparts constitute a pivotal juncture for the precise treatment of cancer, delineating a noteworthy trajectory in the field of targeted therapies. This phenomenon is particularly conspicuous in the domain of nano-drug precision treatment. Despite substantial strides in employing nanoparticles to disrupt ROS for cancer therapy, current strategies continue to grapple with challenges pertaining to efficacy and specificity. One of the primary hurdles lies in the elevated levels of intracellular glutathione (GSH). Presently, predominant methods to mitigate intracellular GSH involve inhibiting its synthesis or promoting GSH efflux. However, a conspicuous gap remains in the absence of a strategy capable of directly and efficiently clearing GSH.

**Methods:**

We initially elucidated the chemical mechanism underpinning oridonin, a diminutive pharmacological agent demonstrated to perturb reactive oxygen species, through its covalent interaction with glutathione. Subsequently, we employed the incorporation of maleimide-liposomes, renowned for their capacity to disrupt the ROS delivery system, to ameliorate the drug’s water solubility and pharmacokinetics, thereby enhancing its ROS-disruptive efficacy. In a pursuit to further refine the targeting for acute myeloid leukemia (AML), we harnessed the maleic imide and thiol reaction mechanism, facilitating the coupling of Toll-like receptor 2 (TLR2) peptides to the liposomes’ surface *via* maleic imide. This strategic approach offers a novel method for the precise removal of GSH, and its enhancement endeavors are directed towards fortifying the precision and efficacy of the drug’s impact on AML targets.

**Results:**

We demonstrated that this peptide-liposome-small molecule machinery targets AML and consequently induces cell apoptosis both in vitro and in vivo through three disparate mechanisms: (I) Oridonin, as a Michael acceptor molecule, inhibits GSH function through covalent bonding, triggering an initial imbalance of oxidative stress. (II) Maleimide further induces GSH exhaustion, aggravating redox imbalance as a complementary augment with oridonin. (III) Peptide targets TLR2, enhances the directivity and enrichment of oridonin within AML cells.

**Conclusion:**

The rationally designed nanocomplex provides a ROS drug enhancement and targeted delivery platform, representing a potential solution by disrupting redox balance for AML therapy.

**Supplementary Information:**

The online version contains supplementary material available at 10.1186/s12951-024-02574-6.

## Introduction

The attainment of successful cancer therapy continues to encounter formidable challenges. An ideal treatment necessitates the precise targeting and elimination of cancer cells while sparing normal cells [1]. The intracellular redox state exists in a dynamic equilibrium [2], orchestrated by controlled reactive oxygen species (ROS) production and elimination [3, 4]. Cancer cells, in their rapid progression, generate substantial ROS to support their advancement [5]. However, an excessive ROS level, commonly referred to as oxidative stress, induces damage to deoxyribonucleic acid (DNA), proteins, and lipids, ultimately culminating in cell death [3, 6]. In response to oxidative stress, the antioxidative system, capable of scavenging ROS, undergoes upregulation as a defense mechanism. Glutathione (GSH) holds significance as a key component of the cellular antioxidative system [7, 8]. In cancer cells, a heightened GSH level is indispensable for scavenging excessive ROS and detoxifying xenobiotics, rendering it a potential target for cancer therapy [9]. The loss of GSH disrupts redox homeostasis, leading to ROS accumulation and, subsequently, cellular dysfunction and death [10]. Notably, cancer cells, with their elevated oxidative stress, exhibit heightened sensitivity to GSH deficiency, presenting a critical vulnerability exploitable in cancer therapy [11, 12].

Nano-strategies for cancer treatment, focused on disrupting ROS, have undergone comprehensive development [13–15]. Within this realm, approaches dedicated to GSH depletion primarily encompass severing the supply of raw materials for GSH synthesis, inhibiting GSH synthesis, depleting GSH reserves, and facilitating GSH efflux [16]. Despite these advancements, a significant challenge persists in the inability to directly target GSH. The efficiency of GSH clearance remains suboptimal, and specificity is not robust enough. The exploration and development of novel and precise nano-strategies tailored for targeted GSH clearance hold profound significance for cancer treatment, particularly in the context of acute myeloid leukemia (AML) [17]. AML is the most frequent leukemia in adults and among the most lethal [18]. AML cells, due to their hypoxic microenvironment in the bone marrow, are more susceptible to ROS than other cancer cells [19, 20]. It has been demonstrated that increasing ROS could easily result in dysfunctions of proliferation or differentiation in AML, and could be an effective strategy for overcoming drug resistance in leukemia [21]. Therefore, depleting GSH to modulate ROS level holds significant promise as an effective therapeutic strategy for treating AML [9, 22, 23].

In this investigation, we initially identified oridonin as a direct covalent inhibitor of GSH, setting it apart from other drugs that disrupt ROS by targeting proteins both upstream and downstream of GSH. However, the clinical advancement of oridonin faced limitations [24, 25], stemming not only from its unclear molecular mechanisms but also due to hydrophobicity and deficient targeting capabilities [26–29]. To address these challenges and elevate oridonin’s therapeutic potential, we opted for liposomes comprising maleimide polyethylene glycol (PEG) for nano-delivery [30, 31]. Diverging from liposomes composed of conventional PEG, those formed by maleimide PEG not only encompass the inherent characteristics of liposomes but also possess the unique ability of depleting GSH upon cellular entry [11]. By selecting maleimide PEG liposomes, we not only resolved the pharmacokinetic hurdles associated with oridonin but also amplified its impact on GSH depletion. Furthermore, the incorporation of maleimide introduced a reactive group for targeting peptides [32]. Expanding on this foundation, we introduced a Toll-like receptor 2 (TLR2) targeting peptide [33, 34]. TLR2, a key member of the toll-like receptor family, is recognized for its ability to bind various natural ligands and can participate its involvement in the progression of diverse diseases [35, 36]. Notably, extensive research has highlighted TLR2’s pronounced expression in various diseases, including cancers [37, 38], autoimmune diseases [39], cardiovascular disorders [40], etc. In the realm of hematological malignancies, Li et al. have revealed a significantly elevated expression of TLR2 in AML [33], compared to chronic myeloid leukemia (CML), acute lymphocytic leukemia (ALL), and chronic lymphocytic leukemia (CLL). Moreover, a TLR2-targeting peptide (His-Leu-Tyr-Val-Ser-Pro-Trp) *via* phage display was identified, which exhibits specific binding to TLR2. Based on these findings, we ultimately culminate a rational design of a liposomal apparatus integrating oridonin, maleimide, and TLR2 targeting peptide for GSH depletion and specific cancer cells delivery.

The in vitro studies demonstrated that we designed liposome complex could penetrate faster and more specifically into AML cells, and trigger more efficiently the ROS elevation and GSH depletion thus result in AML cell apoptosis. Both in vivo and ex vivo AML models demonstrated that we designed liposome complex prolonged the AML cells-bearing mice’s survival significantly, got enriched and retained longer in AML cells. In addition, it did not provoke non-specific toxicity in normal cells and organs, confirming its safety for AML therapy. Taken together, the peptide-liposome-ROS triggering drug complex that incorporated with maleimide we designed provides a ROS drug enhancement and targeted delivery platform, representing a potential solution for AML therapy.

## Methods

### Materials Required for Drug Preparation

Soybean lecithin, DSPE-PEG2000 and DSPE-PEG2000-Maleimide was purchased from Xi’an Ruixi Biological Technology Co., Ltd, oridonin from MCE®, and DCM from Sigma-Aldrich®. TLR2 pep-Cys was chemically synthesized on solid phase. P-methyl-BHA (MBHA) resin was purchased from Applied Biosystems®, Boc protected amino acids were purchased from Peptides Institute®. Other reagents for peptide synthesis and cleavage, including GSH, dichloromethane (DCM), N, N-dimethylformamide (DMF), acetonitrile, Tris-(2-carboxyethyl) phosphine (TCEP), triisopropylsilane (TIPS), N, N-diisopropylethylamine (DIEA), p-cresol and guanidine hydrochloride (GuHCl) were purchased from Sigma-Aldrich®. Trifluoroacetic acid (TFA) was purchased from Halocarbon®.

### Maleimide Doped Oridonin-Liposome Preparation

Soybean lecithin, DSPE-PEG2000 or DSPE-PEG2000-Maleimide, and oridonin were mixed at a mass ratio of 10:2:1. This mixture was then dissolved in dichloromethane (DCM). After fully evaporating, distillated ddH_2_O was added subsequently, and sonicated in the water bath for 10 ~ 20 min, resulting in a semi-transparent, white suspension. The final products were designated as orid-liposome and orid-liposome-MAL, respectively. Subsequently, orid-liposome-MAL were divided equally into two parts. One part was used for subsequent cell experiments, and the other for preparing targeted orid-liposome.

### Conjugation of TLR2 Pep to Maleimide Doped Oridonin-Liposome

A cysteine (Cys) residue was synthetically appended to the C-terminal of the TLR2 targeting peptide, resulting in an amino acid sequence represented as His-Leu-Tyr-Val-Ser-Pro-Trp-Cys. TLR2 targeting peptide was co-incubated and conjugated to the orid-liposome-MAL. The drugs named as 1:1 and 5:1 differed only in the amount of TLR2 pep. TLR2 pep-orid-liposome 1:1 represents the molar ratio of 1:1 for oridonin: TLR2 peptide, whereas 5:1 for TLR2 pep-orid-liposome 5:1. Dialysis was performed to eliminate any excess free peptide post-incubation.

### Morphological Observation

TLR2 pep-orid-liposome was diluted in PBS and then dropped onto the copper net. After complete drying, it was fixed using 2% phosphotungstic acid for 10 min. Subsequent observation was conducted using a transmission electron microscope (TEM) (JEM-100SX).

### Stability and Release Profiles

The prepared TLR2 pep-orid-liposome was exposed to 50% fetal bovine serum (FBS), HCl (pH = 3.0), NaOH (pH = 10.0), and PBS (pH = 7.0) in a 1:1 volume ratio, respectively. After co-incubated for time intervals of 6, 12, 18 and 24 h, high-performance liquid chromatography (HPLC) was employed to evaluate the stability and delineate the release profiles of the liposome.

### Cell Culture

K562, Molm13 cell lines were purchased from DMSZ®. HL60, U937 cell lines were acquired from ATCC®. Additionally, luciferase-Molm13 cell lines were obtained from Nanjing Cobioer Bioscience Co., Ltd. All cell lines were cultured in suspension using RPMI-1640 medium (Gibco®) supplemented 10% FBS (New Zerum®), under the condition of 37 °C and 5% CO_2_.

### Cell Viability Assay

Cell Counting Kit-8 (CCK-8) (Dojindo® Laboratories) assays were employed to evaluate viable cells. Cells were seeded into a 96-well plate at a density of 2 × 10^5^ cells/ml. Treatment groups included liposome, liposome-MAL, oridonin, orid-liposome, orid-liposome-MAL, TLR2 pep-orid-liposome and PBS as a control. After co-culturing for 24–48 h, 10 µl CCK-8 was added into each well. The absorbance was measured *via* a microplate reader. The half maximal inhibitory concentration (IC_50_) of different drugs was analyzed by GraphPad prism software.

### Apoptosis Assay

Cells were seeded and treatment groups were established. After co-incubating for 24 h, cells were harvested by centrifugation and subjected to flow cytometer. Apoptosis detection (Biolegend®) was conducted using a BD FACS Canto II® flow cytometer. Apoptotic cells were identified based on Annexin V+ / PI+, Annexin V+ / PI- or Annexin V- / PI + cells. Data were processed by BD FACS Diva and FlowJo software.

### Immunoblotting Analysis

AML cells were treated with various agents. After 12-hour incubation, cell lysates were collected for analysis. Immunoblotting was conducted to assess the levels of total and cleaved caspase 3. Protein expression through immunoblotting was quantified using Image J software, and statistical analysis was employed for cleaved / total caspase 3. The primary antibody of caspase 3 was purchased from CST®.

### TLR2-binding Test Uing Fluorescence-labeled TLR2 Pep-cys

TLR2 pep-Cys was fluorescently labeled with rhodamine and lyophilized. Cells were co-incubated with rhodamined-TLR2 pep-Cys (2 µM) at room temperature for 2 h. To remove the excess, free, and unbound TLR2 pep, cells were washed with PBS and then fixed with 30% of formaldehyde for 30 min. Fluorescence was detected by flow cytometer using PE channel.

### Up-take Assays by Confocal Microscopy

Cells (K562, HL60, U937, and Molm13) were cultured and treated with rhodamine-labeled TLR2 pep-orid-liposome at a concentration of 2 µM. Uptake of the labeled TLR2 pep-orid-liposome was monitored using a Nikon® confocal microscope. Imaging was conducted at various time points of 5, 20, 40, and 60 min after initiating co-culture with the cells.

### Reductive GSH Level Detection

Cells were and subjected to treatments including PBS, liposome-MAL, oridonin, orid-liposome, orid-liposome-MAL, orid-liposome-TLR2 pep 1:1 and 5:1 at a concentration of 4 µM. After 4-hour incubation, reductive GSH levels were detected using a GSH test kit (Beijing Solarbio®). An equal number of cells were employed for total protein quantification. The final GSH content was calculated using following formula: GSH level = Detected GSH / Total protein.

### Measurement of ROS Level

Cell treatment was same as above. After 6-hour incubation, the ROS Assay Kit -Highly Sensitive DCFH-DA-Reagent (Dojindo® Labratories) were employed for detection. The cells were then incubated with the DCFH probe in a working solution for 30 min and subsequently washed with HBSS. The fluorescence signal of ROS was measured using a flow cytometer, employing the FITC channel.

#### In Vivo Evaluation of Therapeutic Efficacy of Xenograft AML Mouse Model

NSG mice were purchased from Shanghai Model Organisms Center, Inc. Luc-Molm13 cells, at a quantity of 5 × 10^3^, were intravenously injected into NSG mice *via* tail vein to establish a xenograft mouse model. 6 days after injection, 25 mice were randomly divided into five groups (*n* = 5). These groups received intravenous treatments of PBS, oridonin, orid-liposome, orid-liposome-MAL, TLR2 pep-orid-liposome at a dose of 5 mg/kg, or PBS as control every other day. Live imaging was conducted at intervals of 5 ~ 6 days to monitor the progression of the disease. Additionally, survival and body mass of these mice were closely monitored during the treatment.

#### Bio-distribution Test In Vivo

Fluorescent orid-liposome and TLR2 pep-orid-liposome were synthesized with rhodamine labeling for tracking purposes. The AML xenograft model was established by abdominal method, with 1 × 10^6^ HL60 cells intraperitoneally injected in Balb/c nude mice. 21 days post-injection, 24 mice were randomly divided into two groups. These groups received intravenous administration of fluorescent drugs, each at a dosage of 2 mg/kg body weight. At specific time intervals (12, 24, and 48 h), the bio-distribution of orid-liposome or TLR2 pep-orid-liposome in the organs and tumors was quantitatively evaluated by an in vivo optical imaging system (*n* = 3).

#### Toxicity Assessment of Drugs In Vivo

24 mice (Balb/c) were randomly divided into four groups for toxicity assessment. The treatments included PBS, oridonin, orid-liposome, and TLR2 pep-orid-liposome, each administered intravenously at a dose of 5 mg/kg body weight. A total of six doses were administered to the mice, which were then euthanized on the 13th day for analysis. Serum samples were analyzed for liver function markers (Alanine transferase/ALT, Aspartate transferase/AST), renal function markers (Urea nitrogen, Creatinine/Cr), and myocardial enzyme indexes (Creatine Kinase/CK, Creatine Kinase-MB/CK-MB, Lactate Dehydrogenase-L/LDH-L). With a separated batch of animals, established identically, blood cell analysis and hematoxylin-eosin staining (HE) of organs was completed.

### Sorting of AML Primary Cells from Patient Bone Marrow Aspirations

Primary cells from newly diagnosed AML patients’ bone marrow were extracted. The samples are subjected to processes including sedimentation and red blood cell lysis, followed by a brief period of cultivation for further analysis.

### Assessment of TLR2 Expression and Apoptosis

TLR2 expression assay of patient samples were quantified by flow cytometry using PE-Cy7 TLR2 antibody (Biolegend®). Treatments administered to primary AML cells included oridonin, orid-liposome, orid-liposome-MAL, and TLR2 pep-orid-liposome, each at a concentration of 4 µM. After 24 h, flow cytometry was employed to detect apoptosis.

### Sorting of Peripheral Blood Mononuclear Cells (PBMCs) from Healthy Donor

10 ml peripheral blood was provided respectively from two healthy donors. Ficoll-PaqueTM PLUS (Cytiva®) was used to sorting PBMCs.

### Statistical Analysis

For Western Blotting, images were quantified using Image J, and *vinculin* was used for normalization. For cell viability test and GSH detection assay, values of each group were normalized to the control group. For bio-distribution test, fluorescence intensity of each organ was normalized to the cardiac fluorescence values of the corresponding control group. For patient samples, the ratio of apoptotic cells in each treatment group was normalized to control group. The expression of TLR2 above 80% was defined as high expression, while below 80% was defined as low expression. Combination index (CI) was calculated using the CompuSyn software, CI<1.0 was defined as synergism and CI>1.0 was defined as antagonism. These quantification results were presented as the Mean ± SD. Statistical analysis was performed using two-tailed unpaired Student’s *t*-test. One-way ANOVA test followed by Tukey’s *post-hoc* comparison was performed in multiple subgroups. Log-Rank (Mantel-Cox) test was employed to compare the difference in survival analysis. Spearman correlation analysis was used in patient sample experiments. A value of *p* < 0.05 was considered as significantly difference, * indicates *p* < 0.05, ** indicates *p* < 0.01, *** indicates *p* < 0.001, **** indicates *p* < 0.0001, ns stands for not statistically significant. GraphPad 8.0 was used for statistical analysis.

## Results

### Oridonin is a GSH Covalent Inhibitor by Electrophilic Reaction with Thiol Group

**Fig. 1:**
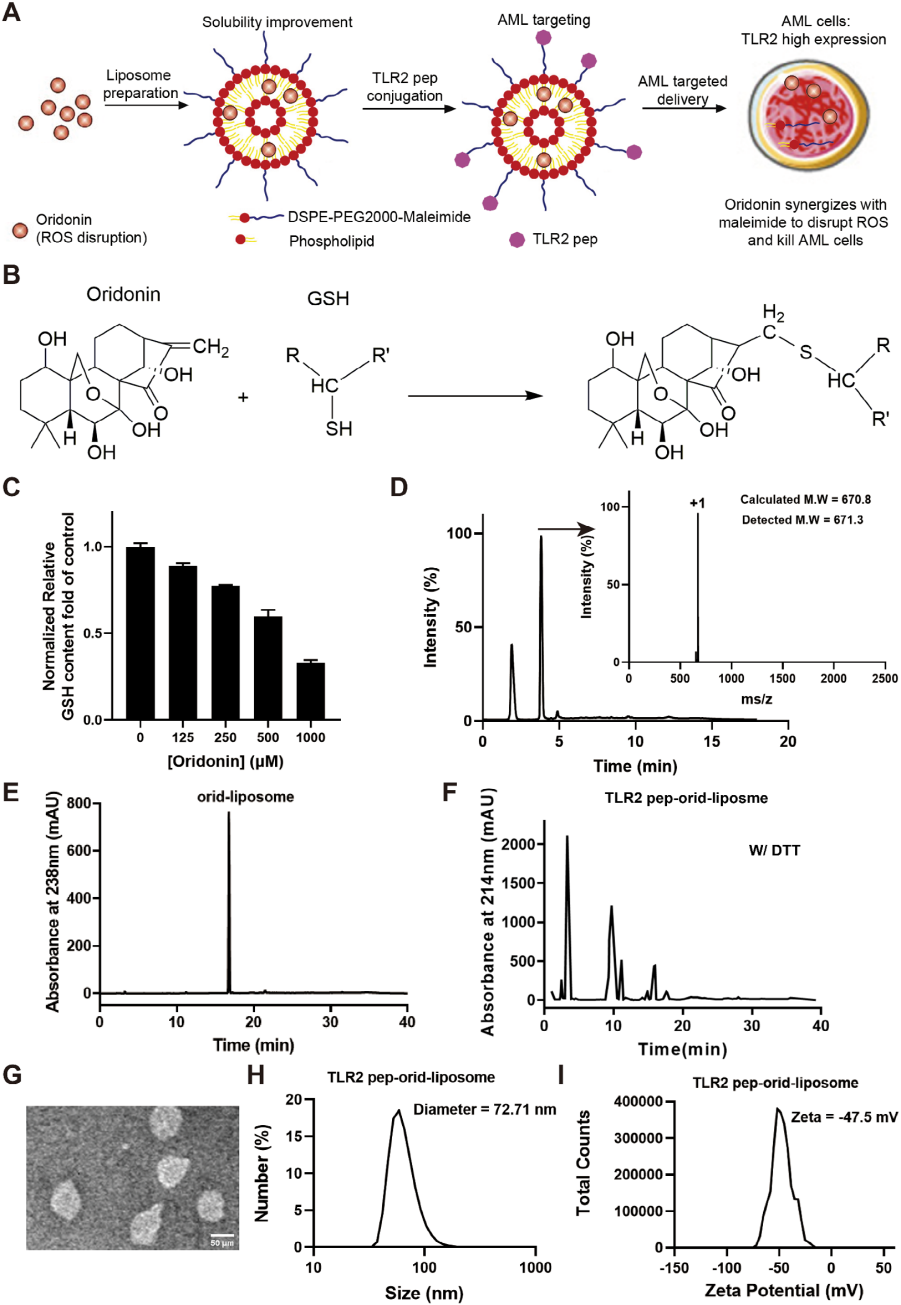
**Oridonin acts as a covalent inhibitor of GSH and the biochemical characterization of TLR2 pep-orid-liposome.** (A) Schematic description of TLR2 pep-orid-liposome design and its potential mechanism illustration for AML therapy. (B) Oridonin reacts with thiol group on reductive GSH through an electrophilic reaction. (C) Reductive GSH detection after a co-incubation of 1 mM GSH with oridonin at different concentration for 4 h. (D) LC/MS analysis of oridonin-GSH after a co-incubation of oridonin and GSH at a molar ratio of 1:1 for 4 h. (E) Orid-liposome analyzed by HPLC with detection UV absorbance at 238 nm. Analytical HPLC was performed on a reversed-phase C18 column (Waters XBridge™ 3.5 μm, 4.6 × 150 mm) at 40 °C. (F) TLR2 pep-orid-liposome 5:1 with 50 mg/ml DTT analyzed by HPLC with detection UV absorbance at 214 nm. (G) TEM image of TLR2 pep-orid-liposome. (H) Hydrodynamic diameter of TLR2 pep-orid-liposome measured by dynamic light scattering. (I) Zeta potential measurement of TLR2 pep-orid-liposome

To establish targeted delivery of oridonin to AML, an initial step involved the construction of liposomes for encapsulating oridonin, aiming to enhance its solubility. Subsequently, TLR2 targeting peptides were coupled with DSPE-PEG2000-maleimide to facilitate the specific delivery to AML cells. Notably, the combined action of oridonin and maleimide significantly enhances the disruption of ROS, aiming to effectively treat AML, as the scheme diagram was shown in Fig. 1A. As depicted in Fig. 1B, the ethylene bond on oridonin represents an electron-vacant site, due to the presence of the *ortho-* and *para-* oxygen atoms. Therefore, oridonin is able to react with thiol group of GSH through an electrophilic reaction, forming C-S bond. Given the crucial role of the thiol group in GSH’s reductive activity, oridonin thus acts as a covalent inhibitor of GSH.

To validate this mechanism, we conducted an in vitro GSH depletion test by co-incubating GSH 1 mM with oridonin at different concentrations for 4 h. Then residual reductive GSH was detected by assessing free thiol content. As shown in Fig. 1C, oridonin effectively inhibited the free thiol of GSH in a concentration-dependent manner. Further elucidation of the mechanism was achieved through liquid chromatography / mass spectrometry (LC/MS) analysis of the oridonin-GSH reaction solution. After reacting oridonin and GSH at a 1:1 molar ratio for 4 h, LC spectra revealed a novel reaction product, indicated by a peak at approximately 4 min. Subsequent MS analysis confirmed that the molecular weight of this product aligns with the anticipated GSH-oridonin adduct (Fig. 1D). Taken together, oridonin is a GSH covalent inhibitor by binding to thiol group of GSH.

### Initial Synthesis and Characterization of TLR2 Pep-Orid-Liposome

Our study initially synthesized of oridonin-loaded liposome, incorporating DSPE-PEG2000-maleimide as needed. As depicted in Fig. 1E and S1A, we quantified oridonin content in these liposomes at 238 nm, with an established standard curve of oridonin *via* HPLC (Figure S1B). To ascertain the loading efficacy of oridonin in TLR2 pep-orid-liposome, batches of products were synthesized, subsequently analyzing the oridonin concentration post-encapsulation through HPLC. The calculated loading efficiency was 9.83 ± 0.15%, indicating successful drug incorporation. Furthermore, we synthesized the TLR2 targeting peptide with an additional Cys residue at the C-terminal to facilitate the binding. The purity and molecular weight of this TLR2 pep-Cys were verified using HPLC and MS, as shown in Figure S2A and S2B. In order to characterize the loading amount of TLR2 peptide for TLR2 pep-orid-liposome, we also established peptide quantification by HPLC using a standard curve (Figure S2C).

As detailed in Figure S3A to C and 1 F, more studies demonstrated that TLR2 peptide release from both TLR2 pep-orid-liposome 1:1 and 5:1 was induced in the presence of 50 mg/ml dithiothreitol (DTT), peaking at 10 min. In the absence of DTT, no free TLR2 peptide was detectable. These results indicate successful loading of TLR2 peptide onto the orid-liposome-MAL *via* covalent conjugation (Michael addition) between the maleimide group of the liposome and the thiol group of the peptide. According to the standard curve of TLR2 peptide, we calculated the peptide loading through peak areas between the two groups of TLR2 pep-orid-liposome 1:1 and 5:1 (W/ DTT) respectively. The molar concentration of orid-liposome-MAL for coupling we applied was 15 µM. TLR2 pep characterized in the 5:1 group was around 2.53 µM (oridonin : TLR2 pep = 15 : 2.53 = 5.93 : 1); while in the 1:1 group, it was 12.47 µM (oridonin : TLR2 pep = 15 : 12.47 = 1.20 : 1). The peptide loading ratio was around 84.3% and 83.3% for TLR2 pep-orid-liposome 5:1 and TLR2 pep-orid-liposome 1:1, respectively. Unless otherwise specified, the TLR2 pep-orid-liposome at the 5:1 molar ratio was referred to as the standard TLR2 pep-orid-liposome in subsequent documentation.

The morphological characteristics of TLR2 pep-conjugated oridonin-loaded liposomes (TLR2 pep-orid-liposome) were investigated using high-resolution electron transmission microscopy (TEM). The image (Fig. 1G) revealed that TLR2 pep-orid-liposome at a 5:1 ratio exhibited a uniform, monodispersed nano-spheric morphology with an average diameter of 78.13 nm, determined from 10 randomly selected particles. Dynamic light scattering in PBS yielded hydrodynamic diameter of 108.2 nm, 81.58 nm, 78.49 nm and 72.71 nm for orid-liposome, orid-liposome-MAL, TLR2 pep-orid-liposome 1:1, and TLR2 pep-orid-liposome 5:1, respectively (Figure S4A to C and 1 H). Additionally, zeta potential values were determined to be -49.7 mV, -46.9 mV, -43.7 mV and − 47.5 mV for the respective formulations (Figure S5A to C and 1I).

Moreover, stability of TLR2 pep-orid-liposome was assessed under varying conditions. Notably, TLR2 pep-orid-liposome was observed instability exclusively when exposed to alkaline conditions (NaOH). Conversely, 24-hour incubation in 50% FBS maintained approximately 70% stability of these nanoparticles (Figure S6A to B). Release profiles were examined following similar procedures as previous experiments, with an additional step of post-centrifugation precipitate evaluation (Figure S6C). The results indicated little precipitation from nanoparticles, with the majority of active ingredients being retained while only a small fraction of the drug was released into the supernatant. These findings align with the notion of physiological stability.

### TLR2 Targeting Peptide Conjugation to Liposome Improved Killing Ability of Oridonin In Vitro

**Fig. 2:**
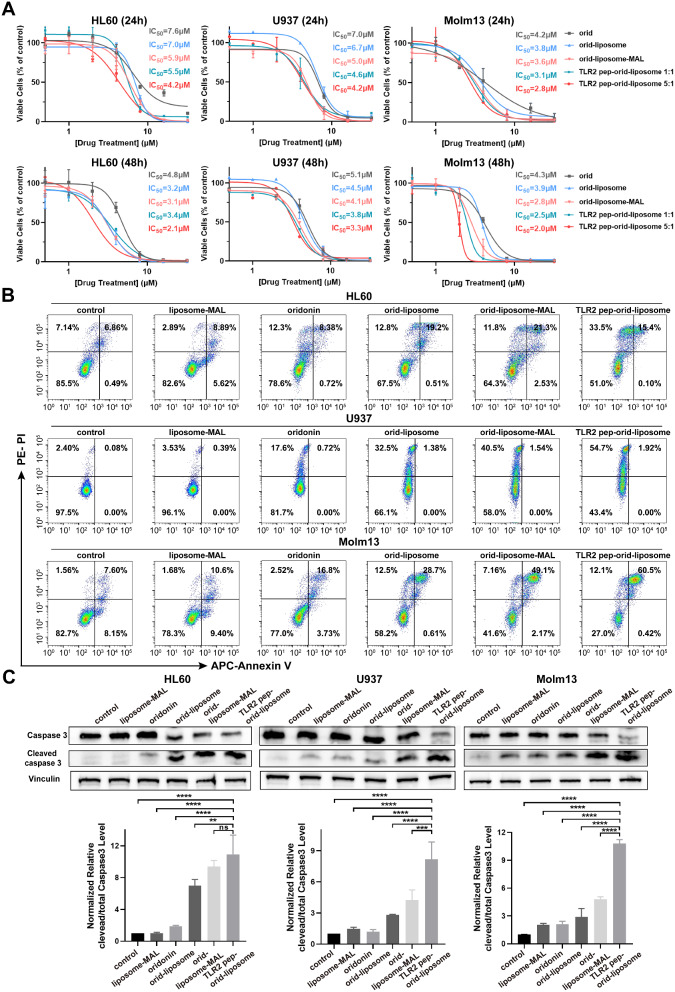
**TLR2 pep-orid-liposome effectively increased the potency of oridonin for AML therapy in vitro**. (A) Viable cells of HL60, U937, and Molm13 cells after 24 and 48 h treatment with varying concentrations of oridonin, orid-liposome, orid-liposome-MAL, TLR2 pep-orid-liposome 1:1 or TLR2 pep-orid-liposome 5:1. (B) Representative data on apoptosis of HL60, U937 and Molm13 after 24-hour treatment with oridonin, orid-liposome, orid-liposome-MAL or TLR2 pep-orid-liposome (4 µM) were analyzed by flow cytometry. (C) Representative western blotting data and statistical results of total and cleaved caspase 3 in HL60, U937 and Molm13 cells after treatment with oridonin, orid-liposome, orid-liposome-MAL or TLR2 pep-orid-liposome for 12 h were shown. (Mean ± SD, *n* = 3; * indicates *p* < 0.05, ** indicates *p* < 0.01, *** indicates *p* < 0.001, **** indicates *p* < 0.0001)

TLR2 pep conjugation, together with maleimide, was expected to improve the AML cell killing ability of oridonin. To determine the most effective maleimide to TLR2 peptide ratio, AML cell lines (HL60, U937 and Molm13) and CML cell line (K562) as control were treated with various liposomal formulations, including orid-liposome, orid-liposome-MAL, TLR2 pep-orid-liposome 1:1, and TLR2 pep-orid-liposome 5:1 over 24 or 48 h. As depicted in Fig. 2A and S7, a concentration- and time- dependent inhibition of cell viability was observed in all AML cell lines and K562. Whereas TLR2 pep alone displayed no inhibitory effect at concentration up to 64 µM (Figure S8). Besides, negative control groups with non-oridonin-loaded liposomes indicated that neither liposome nor liposome-MAL formulations exhibited significant anti-leukemic activity (Figure S9 and S10). Comparative analysis revealed that all oridonin formulations improved the drug’s efficacy to varying extents. Notably, orid-liposome-MAL and TLR2 pep-orid-liposome 5:1, which containing higher free maleimide levels than TLR2 pep-orid-liposome 1:1, demonstrated superior efficacy (Table S1). Considering both target-specific delivery and AML cell killing capacity, TLR2 pep-orid-liposome 5:1 showed to be optimal among different forms of nanoparticles.

To further ascertain the enhanced apoptotic induction by TLR2 pep-orid-liposome in AML cells, we employed Annexin V/PI-based apoptosis analysis. This study encompassed HL60, U937, Molm13 and K562 cells, which treated by liposome-MAL, oridonin, orid-liposome, orid-liposome-MAL or TLR2 pep-orid-liposome at a concentration of 4 µM (Half maximal inhibitory concentration, IC_50_) for 24 h. As anticipated, the results demonstrated that TLR2 pep-orid-liposome notably augmented oridonin’s ability to induce apoptosis in AML cells (Fig. 2B, S11 and S12).

To investigate the intracellular mechanisms of TLR2 pep-orid-liposome, we analyzed the expression levels of total and cleaved caspase 3 in HL60, U937, and Molm13 cells. These cell lines were treated with 4 µM of various formulations for 12 h. As shown in Fig. 2C, western-blotting results revealed that TLR2 pep-orid-liposome significantly induced caspase 3 cleavage. Notably, the ratio of cleaved to total caspase 3 is much higher in the TLR2 pep-orid-liposome group compared to others. This indicates a more rapid cellular penetration of TLR2 pep-orid-liposome and an enhanced ability to induce apoptosis in AML cells *via* a caspase 3-dependent pathway.

### Validation of TLR2 Targeting Peptide Uptake by AML Cells

**Fig. 3:**
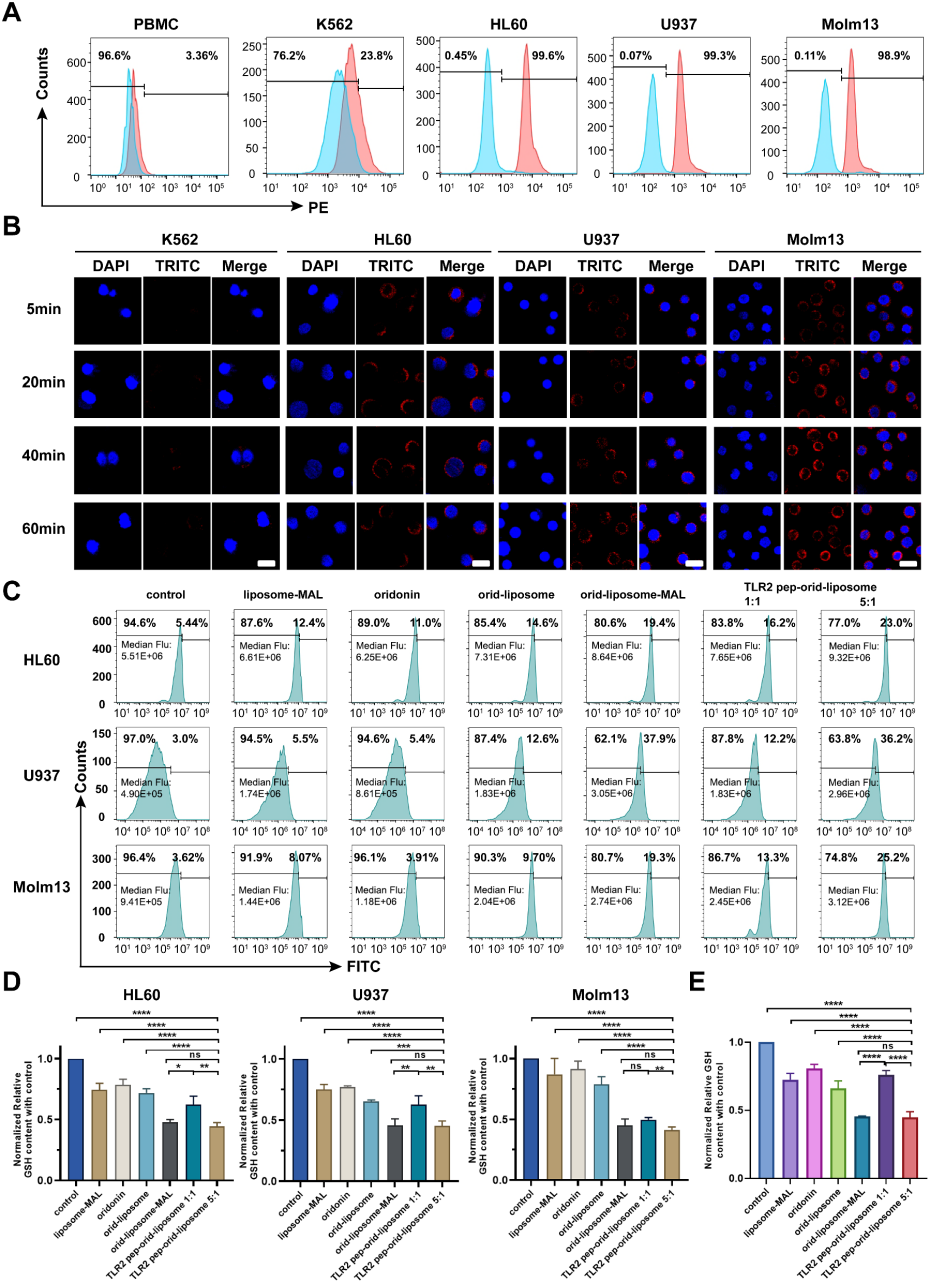
**TLR2 pep-orid-liposome was up-taken by AML cells and effectively stroked the redox balance in AML cells.** (A) Flow cytometry analysis of TLR2 pep-rhodamine uptake by AML cells (HL60, U937, Molm13), CML cells (K562) and PBMC. TLR2 pep-Cys was N-terminally labeled with rhodamine and cultured with cells at 2 µM for 2 h. (B) Confocal microscopy images for TLR2 pep-orid-liposome (rhodamined) uptake, and visualized by a confocal laser scanning microscope (Scale bar = 50 μm). (C) The ROS level detection (median fluorescence is represented in each group) after liposome-MAL, oridonin, orid-liposome, orid-liposome-MAL, TLR2 pep-orid-liposome1:1 and TLR2 pep-orid-liposome 5:1 treatment for 6 h in HL60, U937 and Molm13 cell lines by flow cytometer. (D) The detection of GSH level and the statistical analysis after liposome-MAL, oridonin, orid-liposome, orid-liposome-MAL, TLR2 pep-orid-liposome 1:1 and TLR2 pep-orid-liposome 5:1 treatment for 4 h in HL60, U937 and Molm13. (E) GSH depletion ability detection of liposome-MAL, oridonin, orid-liposome, orid-liposome-MAL, TLR2 pep-orid-liposome 1:1 or TLR2 pep-orid-liposome 5:1 at molecular level. (Mean ± SD, *n* = 3; * indicates *p* < 0.05, ** indicates *p* < 0.01, *** indicates *p* < 0.001, **** indicates *p* < 0.0001, ns stands for not statistically significant)

To investigate how TLR2 pep-orid-liposome improved AML killing ability of oridonin, cellular uptake assays were conducted. Firstly, to ensure the modified TLR2-binding peptide with an extra Cys-residue maintained the TLR2-binding ability, TLR2 pep-Cys was labeled with rhodamine at N-terminus. This labeled peptide was then incubated at a concentration of 2 µM for 2 h with cell lines (K562, HL60, U937 and Molm13) and PBMCs as a control. Subsequent flow cytometry analysis was used to measure cellular fluorescence. As illustrated in Fig. 3A, the modified TLR2 pep-Cys effectively recognized the high TLR2-expressing cell lines HL60, U937, Molm13, and to a lesser extent, the moderately expressing K562 cell line, while showing minimal interaction with TLR2 negative PBMCs. These findings indicate that TLR2 pep-Cys successfully retained its TLR2-specific binding capacity.

To visually track the internalization process of TLR2 pep-guided delivery, TLR2 pep-orid-liposome was prepared using rhodamine-labeled TLR2 pep-Cys to accomplish cellular up-take assays. As shown in Fig. 3B, confocal microscopic images demonstrated rapid labelling of AML cells (HL60, U937, and Molm13) by TLR2 pep-orid-liposome within 5 min of incubation. These liposomes demonstrated efficient cytosolic internalization in a time-dependent manner. Conversely, minimal internalization of TLR2 pep-orid-liposome was observed in K562 cells. Together with the flow cytometry results, these confocal images indicate TLR2 pep-orid-liposome could efficiently facilitate drug delivery into AML cells, with the efficiency being dependent on the level of TLR2 expression.

To further verify whether TLR2 targeting mediates endocytosis, endocytosis inhibitor amiloride (3 mM) was used to treat AML cells 12 h in advance, and then rhodamine-labeled TLR2 pep-orid-liposome was co-incubated for 2 h. The results of flow cytometer detection indicate that amiloride could effectively inhibit TLR2 pep-orid-liposome uptake by HL60, U937 and Molm13 cells, suggesting that TLR2 pep-orid-liposome penetrates TLR2 + cells through receptor-mediated endocytosis (Figure S13).

### Maleimide Doped Liposome Further Triggered ROS Imbalance in AML Cells and Ameliorated the Killing Ability of Oridonin

TLR2 pep-orid-liposome was engineered to trigger GSH exhaustion *via* maleimide, thereby intensifying redox imbalance and enhancing the cytotoxicity against AML cells. To verify the ROS augmentation triggered by this nano-machine, ROS assays were employed using a fluorescent probe in AML cells HL60, U937 and Molm13. As shown in Fig. 3C, both positive percentage and median fluorescence analysis indicated empty liposome-MAL unexpectedly demonstrated superior ROS enhancement than oridonin alone. TLR2 pep-orid-liposome 5:1 showed greater efficacy in inducing ROS than oridonin, orid-liposome or TLR2 pep-orid-liposome 1:1. This augmented efficiency of oridonin in orid-liposome-MAL was not only attributed to its liposomal nature but also to the presence of maleimide. The weaker effect observed with TLR2 pep-orid-liposome 1:1, compared to the 5:1 ratio, and is likely due to a higher occupancy of DSPE-PEG2000-MAL sites by TLR2 pep.

To delve deeper into the mechanism, we performed reduced GSH quantification assays in AML cells, following treatment by liposome-MAL, oridonin, orid-liposome, orid-liposome-MAL, TLR2 pep-orid-liposome 1:1 and TLR2 pep-orid-liposome 5:1. All treatments were administered at an oridonin-equivalent concentration of 4 µM, with PBS serving as the control. This biochemical reaction results in the production of a yellow compound, 2-nitro-5-mercaptobenzoic acid, identifiable at a wavelength of 412 nm. As demonstrated in Fig. 3D, the treatments with orid-liposome, orid-liposome-MAL, and TLR2 pep-orid-liposome 1:1 and TLR2 pep-orid-liposome 5:1 notably depleted reductive GSH within 4 h. This effect was significantly more pronounced compared to the control, oridonin, and liposome-MAL. Among these, orid-liposome-MAL and TLR2 pep-orid-liposome 5:1 exhibited superior efficacy, attributable to the incorporation of maleimide.

At the molecular level, GSH depletion assay was also conducted by co-incubating 1 mM GSH with various compounds: oridonin, liposome-MAL, orid-liposome, orid-liposome-MAL, and orid-liposome-TLR2 pep 1:1 or 5:1, each at a concentration of 1 mM for 4 h. Figure 3E demonstrated that all treatment groups effectively depleted GSH. Notably, orid-liposome-MAL, containing maleimide, was more efficient in GSH depletion compared to orid-liposome. TLR2 pep-orid-liposome 5:1 showed comparable efficacy to orid-liposome-MAL, ranking them as the most effective among all groups. In the case of TLR2 pep-orid-liposome 1:1, majority of maleimide was bond to TLR2 pep, resulting in a GSH depletion capacity similar to that of orid-liposome without maleimide, and thus less efficient than 5:1 variant, which possesses more amount of free maleimide. Taken together, the presence of free maleimide enhances GSH exhaustion. TLR2 pep-orid-liposome 5:1 could simultaneously trigger inverse changes in GSH and ROS, thus further ameliorating AML killing ability of the original ROS targeting drug, oridonin in occurrence.

To validate the synergistic effect at molecular level, separate additions of oridonin and liposome-MAL were made to an excess of GSH solution (1 mM) across concentration gradients. After 4-hour incubation, we quantitatively measured the residual GSH levels. Subsequently, maintaining a constant concentration of liposome-MAL at 1 mM, we continued to add oridonin in concentration gradients to assess its synergistic ability in depleting GSH. The parameter CI was calculated using the CompuSyn software, which is quantifying the interaction between drugs. In the results presented in Table S2, it is evident that the CI is less than 1.0 (CI<1.0 was defined as synergism; CI>1.0 was defined as antagonism) for all concentration gradients, indicating a synergistic interaction between the oridonin and liposome-MAL. However, it is worth noting that our design is not simply the straightforward combination of two drugs. In fact, during the synthesis of the nano-medicine, the mass ratio of orid-liposome-MAL ingredients is fixed, and it mainly involves a change in the form of drug. Therefore, using the CI index or evaluating oridonin, liposome-MAL and orid-liposome-MAL in comparison is not rigorous. Additionally, oridonin induces explicit cytotoxic effects in AML cells, while liposome-MAL does not exhibit the characteristic (Figure S10), which only caused slightly changes in GSH level. Consequently, it was determined an effective strategy through improvement of oridonin by liposome-MAL preparation.

### TLR2 Pep-Orid-Liposome Showed Potent Efficacy for AML Therapy in luc-Molm13 Xenograft NSG Mouse Model In Vivo

**Fig. 4:**
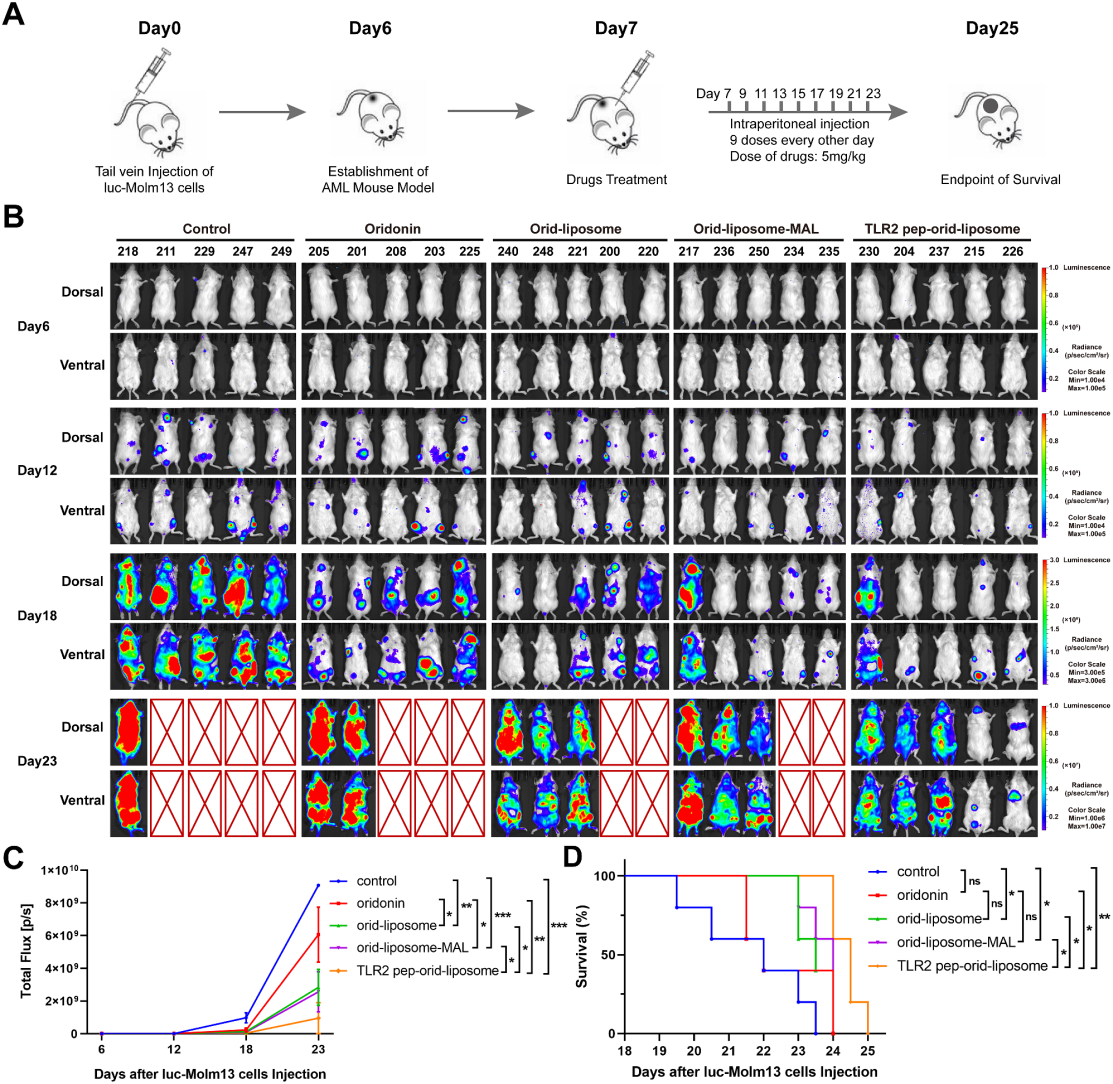
**The TLR2 pep-orid-liposome effectively extended the life span of AML mice.** (A) Schematic diagram of luc-Molm13 xenograft NSG mouse model establishment and drug treatment. 25 tumor-bearing mice were randomly divided into five groups (*n* = 5), and then treated every other d y *via i.p.* injection of PBS (vehicle), oridonin, orid-liposome, orid-liposome-MAL or TLR2 pep-orid-liposome at a dose of 5 mg/kg. (B) Representative fluorescence images of the mice were displayed from each treatment group. (C) Fluorescence intensity in each mouse was determined using Living Image 3.0 Software and GraghPad was employed for statistical profile. (D) Survival curve and analysis in different groups of AML mice. (Mean ± SD, *n* = 5; * indicates *p* < 0.05, ** indicates *p* < 0.01, *** indicates *p* < 0.001, **** indicates *p* < 0.0001, ns stands for not statistically significant)

To evaluate the efficacy of TLR2 pep-orid-liposome in vivo, we established an AML xenograft mouse model using luciferase-Molm13 cells in NSG mice. Mouse model has been verified to be powerful for hematologic tumor research in several studies [41]. Figure 4A illustrates the experimental setup, where 5 × 10^3^ luc-Molm13 cells were injected into NSG mice *via* tail vein. 6 days after injection, 25 mice were randomly divided into five groups (*n* = 5). These groups received intravenous treatments of oridonin, orid-liposome, orid-liposome-MAL, and TLR2 pep-orid-liposome at a dosage of 5 mg/kg (equivalent to the active ingredient of oridonin), with PBS serving as the control, administered every other day. The tumor-bearing mice were monitored by living imaging, and the results were presented in Fig. 4B. Not surprisingly, the statistical outcomes demonstrated that TLR2 pep-orid-liposome exhibited the best efficacy in reducing tumor burden, as evidenced by the total flux (summed up fluorescence of dorsal and ventral) among the 5 groups (Fig. 4C), with a significant difference observed compared to the other treatment groups. These mice survived for a total of 25 days and naturally died, which we defined as the survival endpoint as illustrated in Fig. 4D. Results indicated that TLR2 pep-orid-liposome significantly prolonged the survival of tumor-bearing mice compared to the control group. In contrast, oridonin alone did not demonstrate sufficient efficacy at the same dosage. During the progress, the actual body weight variation of mice was closely monitored. Figure S14 indicates no significant difference in body weight changes across these treatment groups.

### Rhodamined TLR2 Pep-Orid-Liposome Efficiently Accumulated and Retained In Vivo

To explore the bio-distribution of TLR2 pep-conjugated drug, we prepared rhodamine-labeled orid-liposome and TLR2 pep-orid-liposome. The liposome formulation process is identical to the previously described protocol, with the incorporation of fluorescent rhodamine into these formulations. Fluorescent liposomes (rhdamined orid-liposome or TLR2 pep-orid-liposome) were intravenously administered into tumor-bearing Balb/c nude mice, which had been intraperitoneally inoculated with HL60 cells. The bio-distribution of orid-liposome or TLR2 pep-orid-liposome in various organs and tumor was quantitatively evaluated at three distinct time points (12, 24, and 48 h post-injection) by an in vivo optical imaging system (Mean ± SD, *n* = 3). As illustrated in Figure S15, TLR2 pep-orid-liposome reached a maximum concentration in tumors as early as 12 h post-administration, which exhibited greater efficient than that of orid-liposome at 24 h. Besides of the AML tumors, TLR2 pep-orid-liposome predominantly accumulated in the kidney and liver. Notably, significant fluorescence was retained in the liver at 48 h, aligning with the liver’s role in liposome clearance. These observations indicate that in presence of TLR2 targeting peptide, TLR2 pep-orid-liposome is capable more extendedly of accumulating and retaining in AML, thereby efficiently delivering drug to the target site.

To further provide evidence supporting enhanced tumor accumulation of TLR2 pep-orid-liposome, a detailed analysis comparing the fluorescence ratios (tumor/liver) was conducted. The results clearly demonstrate that TLR2 pep-orid-liposome exhibited significantly higher and faster drug accumulation in tumors compared to the orid-liposome group, particularly at the 12-hour (Figure S16). Specifically, the fluorescence ratio for TLR2 pep-orid-liposome was 0.49 ± 0.05, in contrast to 0.08 ± 0.01 for the orid-liposome group at this time point. These findings robustly support the notion that TLR2 pep-orid-liposome facilitates delivery of the drug specifically to tumor sites, thus minimizing off-target effects in the liver while maximizing therapeutic efficacy against AML cancer cells.

### TLR2 Pep-Orid-Liposome Treatment Not Showed Obvious Toxicity In Vivo

**Fig. 5:**
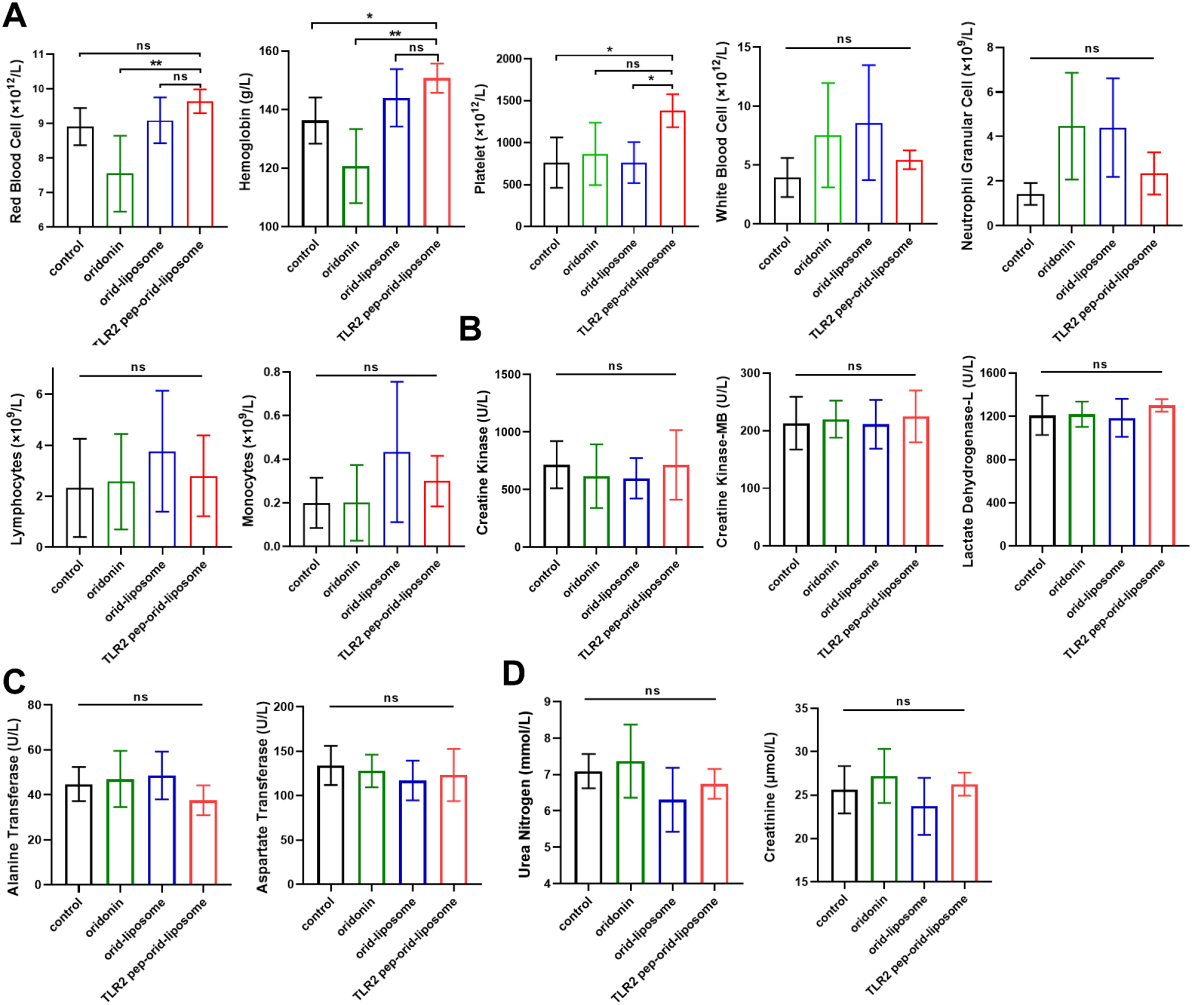
**Toxicity evaluation of oridonin, orid-liposome, or TLR2 pep-orid-liposome treatment in vivo**. (A) Hematological toxicity analysis was conducted following each drug treatment and assessed by blood cells examination in AML xenograft mouse model. (Mean ± SD, *n* = 4). (B) Cardiac toxicity (Creatine Kinase, Creatine Kinase-MB, Lactate Dehydrogenase-L) analysis in normal Balb/c mouse after drug treatments. (C) (D) Liver function (Alanine Transferase/ALT, Aspartate Transferase/AST) and renal function (Urea Nitrogen, Creatinine) detection in normal Balb/c mouse after each drug treatment. (Mean ± SD, *n* = 6; * indicates *p* < 0.05, ** indicates *p* < 0.01, ns stands for not statistically significant)

Bone marrow suppression is a frequent adverse event associated with many chemotherapeutic drugs. To evaluate the hematological toxicity of TLR2 pep-orid-liposome, blood cell analysis was performed on peripheral whole blood from treated mice. This analysis focused on monitoring levels of hemoglobin (Hb), white blood cells (WBC), and platelets (PLT). The findings indicated that, at the administered dosage, TLR2 pep-orid-liposome did not cause significant hematological inhibition when compared to the control group, as shown in Fig. 5A. Moreover, it demonstrated enhanced efficacy in mitigating reductions in Hb and PLT levels.

To assess potential organ toxicity, an extensive toxicity analysis was carried out. TLR2 pep-orid-liposome and various control groups (*n* = 6) were administered to Balb/c mice every other day for a total of six doses. Following this regimen, a comprehensive evaluation of hepatic, renal, and myocardial toxicity was conducted. The dosage for each drug across all groups was standardized at 5 mg/kg to ensure consistency in the assessment. Specifically, the obtained serum samples were subjected to rigorous analysis for myocardial enzymes (CK, CK-MB, and LDH-L), liver function (ALT, AST) and renal function (Urea nitrogen, Cr). As presented in Fig. 5B to D, the results clearly demonstrated that TLR2 pep-orid-liposome did not exhibit any evident toxicity. Moreover, HE staining analysis confirmed that no significant organ toxicity associated with this drug (Figure S17).

### TLR2 Pep-Orid-Liposome Induced Apoptosis of AML Patient Primary Cells Ex Vivo

**Fig. 6:**
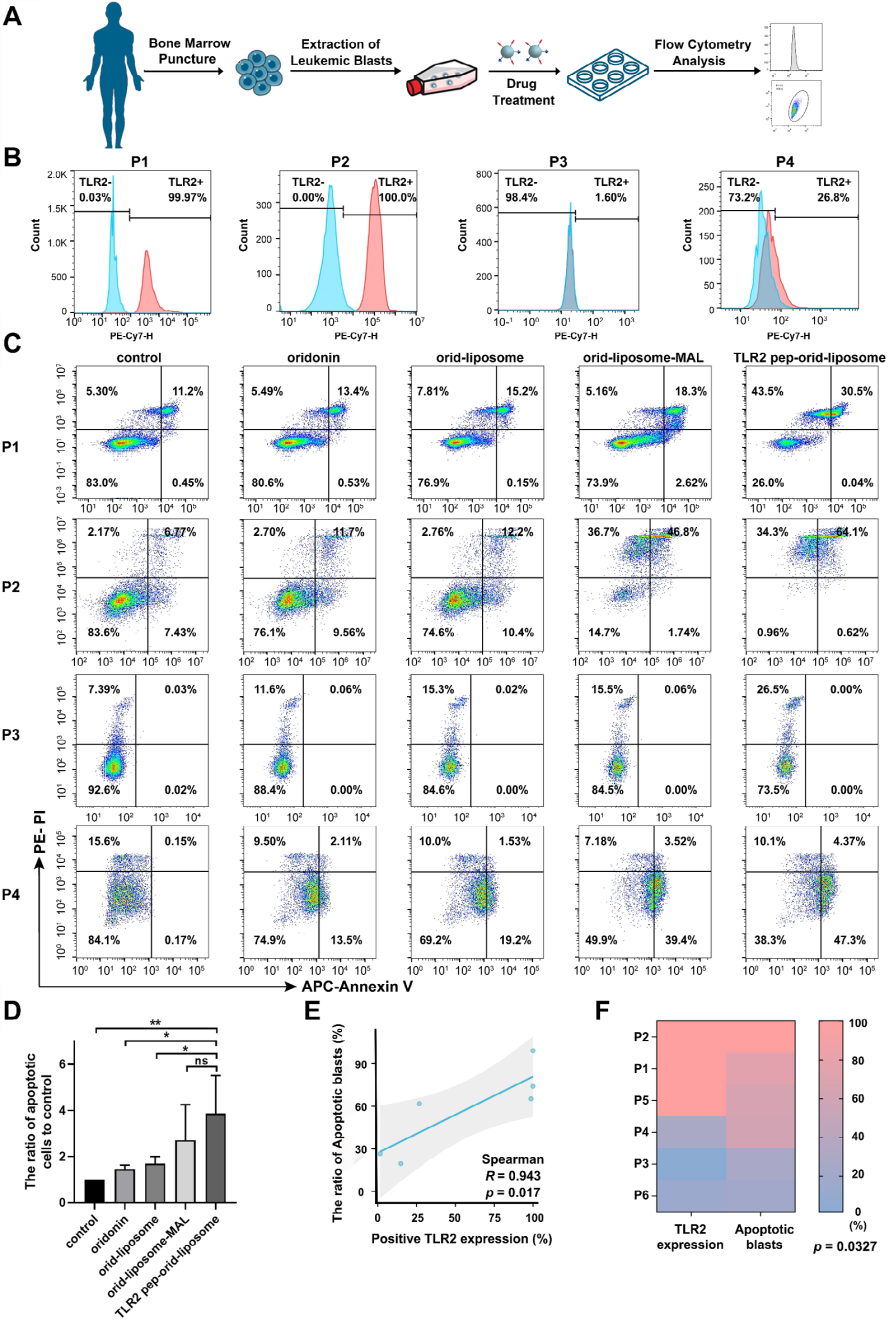
**The ex vivo sensitivity of primary cells from patients with AML to oridonin, orid-liposome, orid-liposome-MAL, or TLR2 pep-orid-liposome.** (A) Schematic of the treatment for newly diagnosed AML patient specimens. (B) The expression of TLR2 in AML patient blasts was detected by flow cytometry. (C) Apoptosis test of AML specimens after 24-hour treatment with oridonin, orid-liposome, orid-liposome-MAL, or TLR2 pep-orid-liposome (4 µM). (D) Statistical analysis of AML cells after treatment of different oridonin variants. (E) Statistical results of Spearman correlation between positive TLR2 expression level and ratio of apoptotic blasts (*R* = 0.943, *p* = 0.017). (F) Heatmap of the correlation between TLR2 expression and apoptotic blasts (*p* = 0.0327). (Mean ± SD, *n* = 6; * indicates *p* < 0.05, ** indicates *p* < 0.01, ns stands for not statistically significant)

Ex vivo cytotoxicity assays utilizing primary leukemic cells isolated from patients provide a valuable approach for assessing the therapeutic efficacy of antitumor agents, especially for hematological malignancies. Primary leukemic cells were obtained from *de novo* diagnosed AML patients; specific diagram had been presented in Fig. 6A. The basic information was presented in Table S3. The blasts were treated with PBS, oridonin, orid-liposome, orid-liposome-MAL and TLR2 pep-orid-liposome at a concentration of 4 µM for 24 h. The expression of TLR2 was detected by flow cytometry, and the positive expression level was counted. It was defined that the expression of TLR2 above 80% was defined as high expression, while below 80% was defined as low expression (Fig. 6B and S18). As depicted in Fig. 6C, S19 and 6D, in all patient samples, oridonin exhibited a certain level of killing effect compared to the control group. However, TLR2 pep-orid-liposome significantly enhanced the killing effect of oridonin. These results, obtained from freshly patient samples, suggested the potential for achieving a clinical response in AML patients with high TLR2 expression using TLR2 pep-orid-liposome. Notably, the ratio of apoptotic cells in the TLR2 pep-orid-liposome group was significantly and positively associated with the expression level of TLR2 according to statistical results. As indicated in Fig. 6E, the Spearman correlation coefficient between TLR2 positive expression level and the proportion of apoptotic blasts was calculated (*R* = 0.943, *p* = 0.017). Furthermore, heatmap in Fig. 6F also indicated their close relation and significant differences (*p* = 0.0327).

Moreover, to evaluate the potential cyto-toxicity of TLR2 pep-orid-liposome, we performed an apoptosis analysis following drug treatments on PBMCs from two healthy donors (Table S4). As expected in Figure S20, our results showed a modest induction of cell death in normal cells, indicating a favorable safety profile for the targeting drug with minimal impact on healthy cells.

## Discussion

**Fig. 7:**
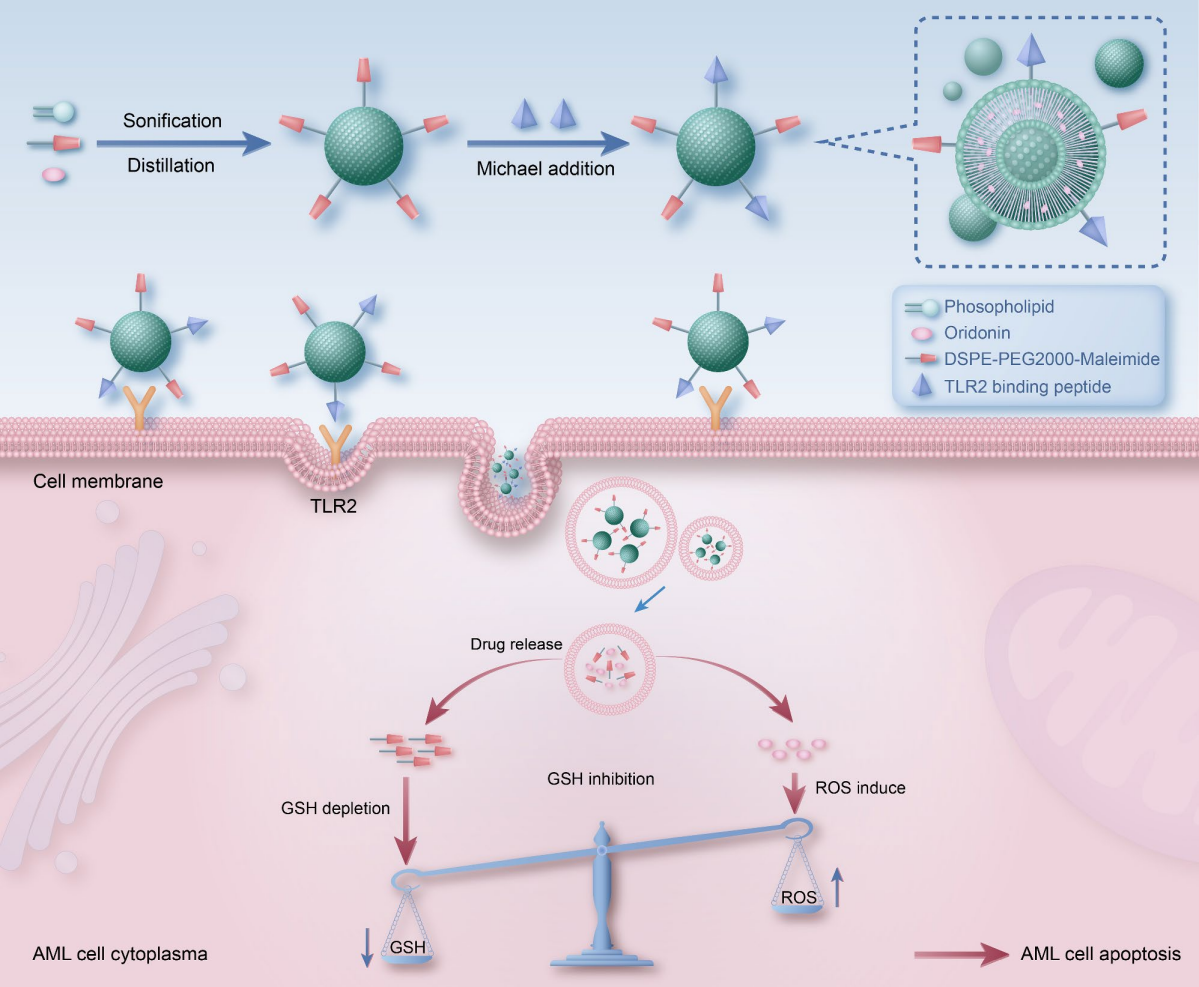
**Schematic diagram of TLR2 pep-oridonin-liposome for targeted molecular therapy of acute myeloid leukemia.** The TLR2 targeting peptide (TLR2 pep) was reversibly conjugated to oridonin liposomes through Michael addition reaction. These nanoparticles can be enriched on and get internalized through TLR2 targeting on AML cell surface. Once in cytosol, TLR2 pep-orid-liposome releases oridonin, which induces ROS, while maleimide exhausts cellular reductive glutathione to enhance redox imbalance and kill AML cells

In this study, we have developed a novel AML-specific drug-delivery system, TLR2 pep conjugated oridonin-liposome. The design strategy and mechanism were outlined in Fig. 7. Firstly, oridonin was encapsulated in liposomes while maleimide was doped using DSPE-PEG2000-maleimide. TLR2 pep was chemically synthesized with an additional cysteine residue at the C-terminal. TLR2 pep-Cys was subsequently conjugated onto orid-liposome through reversible Michael addition reaction, through a cysteine residue between maleimide and thiol group. These obtained nanoparticles, namely TLR2 pep-orid-liposome, loaded of oridonin and TLR2 pep can be enriched on and get internalized in AML cells. Once in the cytosol, TLR2 pep-orid-liposome releases oridonin, which induces ROS elevation. Meanwhile, maleimide will exhaust cellular GSH to further enhance the ROS generation. Through the efficiency improvement mechanism, these nanoparticles will be able to enhance intracellular redox imbalance and efficiently kill AML cells. Taken together, our *in vitro, in vivo* and ex vivo models provide compelling evidence regarding the therapeutic effect and safety profile of TLR2 pep-orid-liposome as a promising clinical treatment for AML.

Efforts are needed and being made by scientists to find more efficient while less toxic therapeutics for acute myeloid leukemia, as well as for cancers in general [42]. Oridonin originally extracted from *Rabdosia rubescens* have shown great anti-leukemic potential as candidate drug. In this work, we firstly clarified that oridonin is a Michael acceptor molecule, reacting to thiol group of GSH by an electrophilic addition and covalent bonding. Analogously, KRAS inhibitors (Sotorasib) targeting the G12C mutation and eprenetapopt (APR-246), a novel drug for *Tp53*-mutant MDS and AML, both are representatives of this molecules. These drugs have received approval by FDA and achieved successful entry to market, bolstering our confidence in the clinical potential of our oridonin.

To resolve the poor hydro-solubility problem, oridonin was encapsulated in form of liposome. To further improve its pharmacological properties such as efficacy and retaining, we have rationally designed a maleimide-doped liposome while synthesis. These maleimide functional groups play dual roles, that permitting a Toll-like receptor 2 binding peptide conjugation in order to improve cancer cell specific delivery, in the other hand, once in cytosol, maleimide efficiency improve oridonin’s redox imbalance promoting ability by simultaneous GSH depletion. The changes in GSH levels induced by such stress are instantaneous and drastic processes, while the generation and replenishment of GSH require a lengthy process, due to involving the rate-limiting indicator glutathione synthetase [43]. Consequently, this design aggravates the redox imbalance, causing a rapid increase in ROS in the short term in vitro, ultimately leading to the downstream signaling pathway activation and apoptosis of AML cells.

Targeted therapy remains a key strategy in treating hematological malignancies. Herein, we rationally designed the targeting nano-complex TLR2 pep-orid-liposome, and compared the differences among these variants in detail. In contrast to oridonin alone, orid-liposome has a better efficacy due to its better water solubility. Orid-liposome-MAL showed stronger killing effect than orid-liposome under the action of enhancing GSH depletion in vitro. As for the culture environment in vitro, where only AML cells are present, orid-liposome-MAL can non-competitively enter all AML cells, causing REDOX imbalance and exerting its anti-tumor effects. Therefore, our study emphasizes that maleimide exhibits a synergistic effect with GSH depletion in vitro. However, the in vivo environment is more complex with the presence of numerous other non-tumor cells. The maleimide group enters into all cells indiscriminately, which results in weaker cytotoxic effects, due to lacking of specific targeting. However, the TLR2 pep-orid-liposome showed the best killing effect due to AML specific delivery *via* TLR2 peptide. We have demonstrated in vitro as well as in vivo, such nano-delivering machinery has indeed improved the therapeutic efficacy and AML-specific killing of oridonin. Simultaneously, we proved the bio-distribution and AML cell retaining ability were greatly ameliorated in vivo, paving the road to clinical development of oridonin for AML therapy.

Furthermore, we validated on drug penetration into cells, putting forward that the internalization of our nano-machinery may be mediated by TLR2. However, the detailed endocytosis mechanism remains unclear. The cellular uptake of nanoparticles involves various endocytic pathways, including phagocytosis, macropinocytosis, clathrin- and caveolin-mediated endocytosis. The specific pathway utilized depends on several factors, such as specific cell type, cellular milieu, the material properties of size, shape, surface coating as well as electrical charge, and the interactions established with the cellular components [44]. Hence, further investigations are necessary to gain a deeper understanding of the endocytic uptake mechanisms associated with liposome-targeting nanoparticles. These studies will facilitate the exploitation of this nanomedicine strategy for potential applications in treating various diseases.

It is worth that anti-cancer therapy development, even all pharmaceutical developments, represents a multi-disciplinary coordination from fundamental science, physicians to pharmaceutical industries, and requires mount of investment resources. Only a minor part can stand out from vast selection, and will be able to enter in clinical trial, thus representing a precious treasure for medical development and concealed hope for patients. Whereas, hydrophobic small molecules frequently failed on the road toward clinical use, due to variable and complexed problems including bio-availability, and swift clearance, which in turn because of their physicochemical nature. Therefore, using FDA approved nanotechnology, chemical adjuvant and bio-degradable and low toxic peptide conjugation, such as liposomes, maleimide, which frequently regarded as ADC linker and peptide, respectively, to rescue candidate drugs, offering a new strategy for drug development with potential in clinical use.

## Conclusion

The modified nano-product (TLR2 pep-orid-liposome) with structural encapsulation, efficiency enhancement and specific targeting of oridonin demonstrate the potential to a candidate for AML therapeutics. It provides a ROS drug enhancement and targeted delivery platform as well, which is widely applicable to other hydrophobic small molecules.

### Electronic supplementary material

Below is the link to the electronic supplementary material.

Supplementary Material 1

## Data Availability

No datasets were generated or analysed during the current study.
