# Supplementary material for: Glutathione-depleting Liposome Adjuvant for Augmenting the Efficacy of a Glutathione Covalent Inhibitor Oridonin for Acute Myeloid Leukemia Therapy
Source: J Nanobiotechnology. 2024 May 30;22:299. doi: 10.1186/s12951-024-02574-6 (PMC11137913; doi:10.1186/s12951-024-02574-6)
Supplement: Supplementary file 1 — Supplementary Material 1 [file 12951_2024_2574_MOESM1_ESM.doc]

**Supplemental Information**

**Glutathione-depleting liposome adjuvant for augmenting the efficacy of a Glutathione covalent inhibitor oridonin for acute myeloid leukemia therapy**

*Yi Liu ^1,†^, Xiaoning Wang ^1,†^, Hui Feng ^1,†^, Xinyan Li ^1^, Runyu Yang ^1^, Mengyao Zhang ^1^, Yue Du ^1^, Ruimin Liu ^1^, Minna Luo ^1^, Zhiyi Li ^1^, Bo Liu ^2^, Jincheng Wang ^1^, Wenjuan Wang ^1^, Feifei An ^3,*^, Fan Niu ^1,*^, Pengcheng He ^1,*^*

^1^ Department of Hematology, The First Affiliated Hospital of Xi’an Jiaotong University, No. 277 Yanta West Road, Xi'an, 710061 Shaanxi, China

^2^ Department of Urology, The First Affiliated Hospital of Xi’an Jiaotong University, No. 277 Yanta West Road, Xi'an, 710061 Shaanxi, China

^3^ School of Public Health, Health Science Center, Xi'an Jiaotong University, No.76 Yanta West Road, Xi'an, 710061 Shaanxi, China

E-mail: [hepengcheng@xjtu.edu.cn](mailto:hepengcheng@xjtu.edu.cn); [niufan@xjtufh.edu.cn](mailto:niufan@xjtufh.edu.cn); anfeifei@xjtu.edu.cn

† These authors contributed equally to this work.

* Corresponding authors.

**Keywords:** Oridonin; Peptide-based drug delivery; Glutathione inhibitor; ROS; AML therapeutics

**Figure S1.**

**
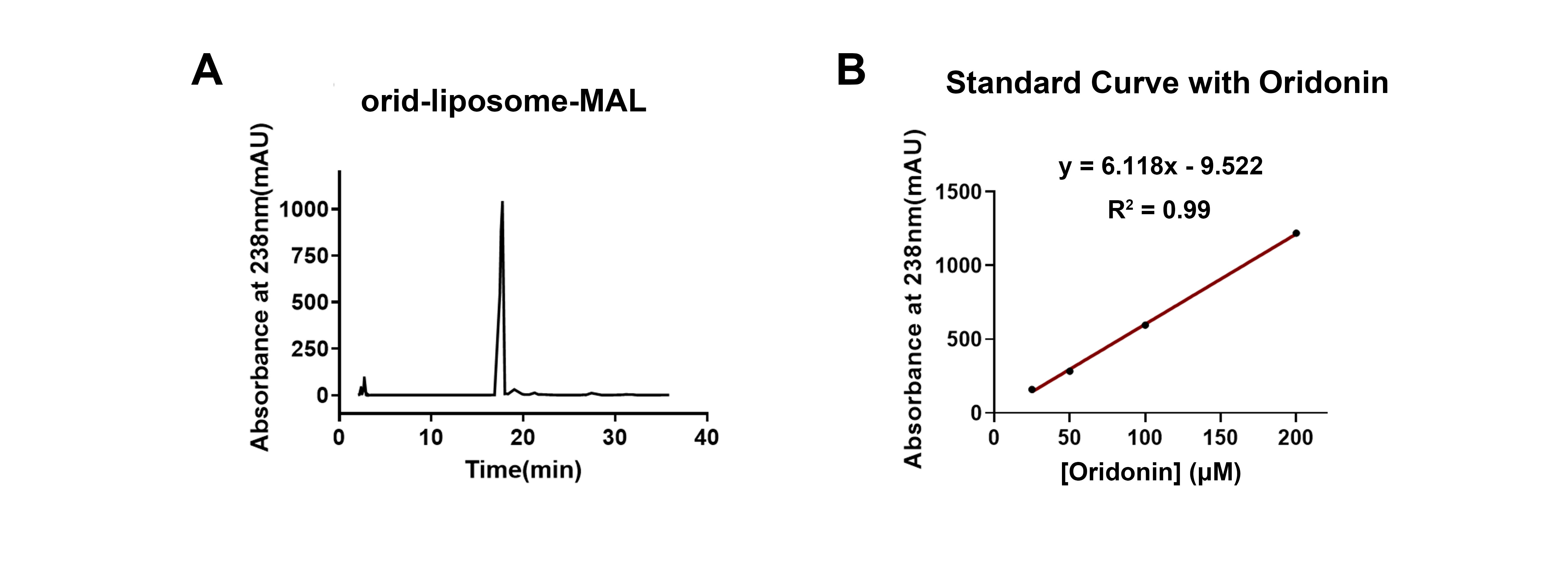
Figure S1.** **Characterization and quantification of oridonin loading in liposomes.** (A) Orid-liposome-MAL analyzed by HPLC with detection UV absorbance at 238 nm. Analytical HPLC was performed on a reversed-phase C18 column (Waters XBridge™ 3.5 μm, 4.6 × 150 mm) at 40°C. (B) Standard curve of oridonin, analyzed by HPLC with detection UV absorbance at 238 nm and calculated from HPLC peak area.

**Figure S2.**

**
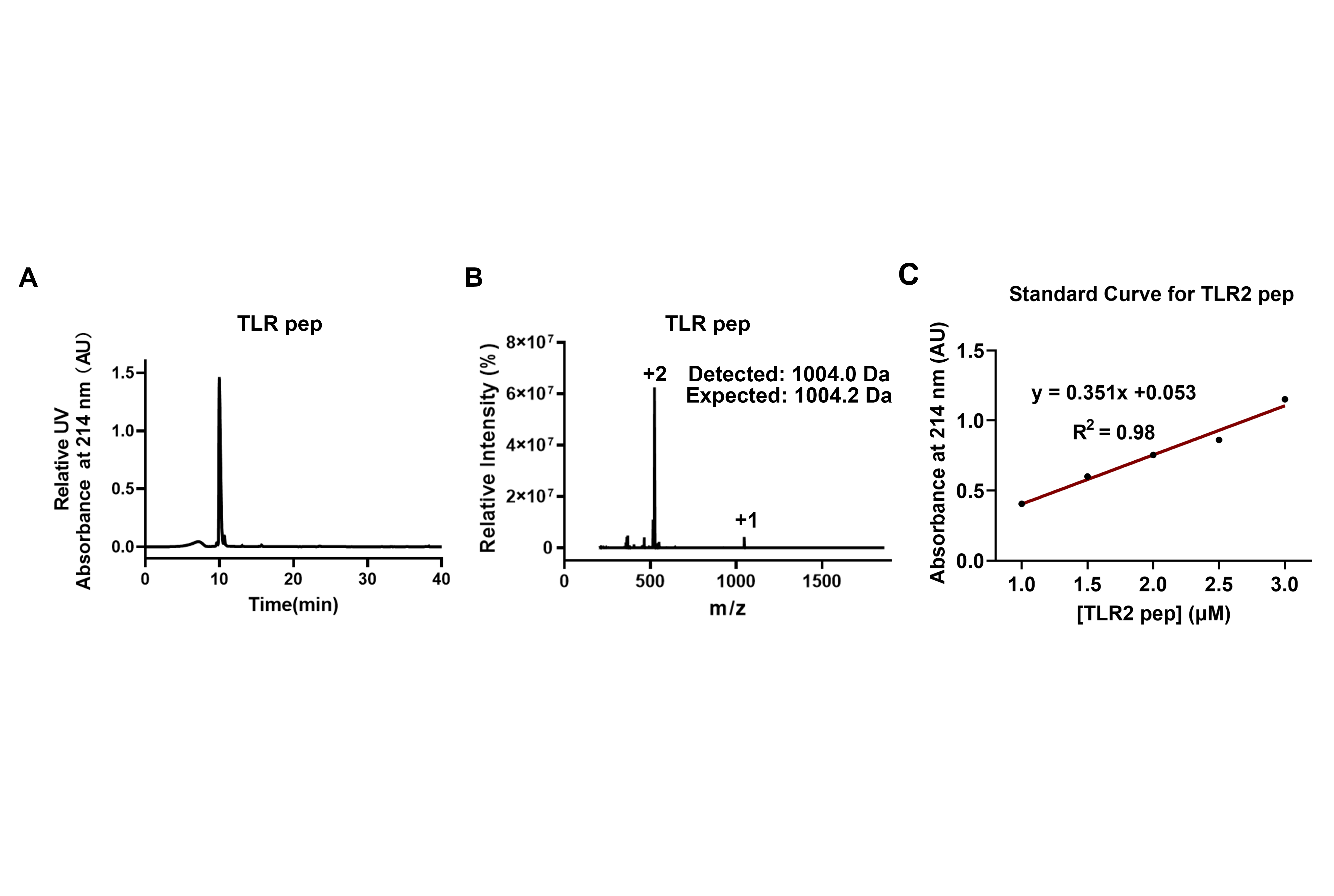
Figure S2. Characterization of synthesized TLR2 targeting peptide with a Cys residue TLR2 pep-Cys.** (A) TLR2 pep-Cys analyzed by analytical HPLC with detection UV absorbance at 214 nm on a reversed-phase C18 column (Waters XBridge™ 3.5 μm, 4.6 × 150 mm) at 40 °C. (B) TLR2 pep-Cys analyzed by electrospray ionization mass spectrometry (ESI-MS). (C) Standard curve of TLR2 pep-Cys, analyzed by HPLC with detection UV absorbance at 214 nm and calculated from HPLC peak area.

**Figure S3.**

**
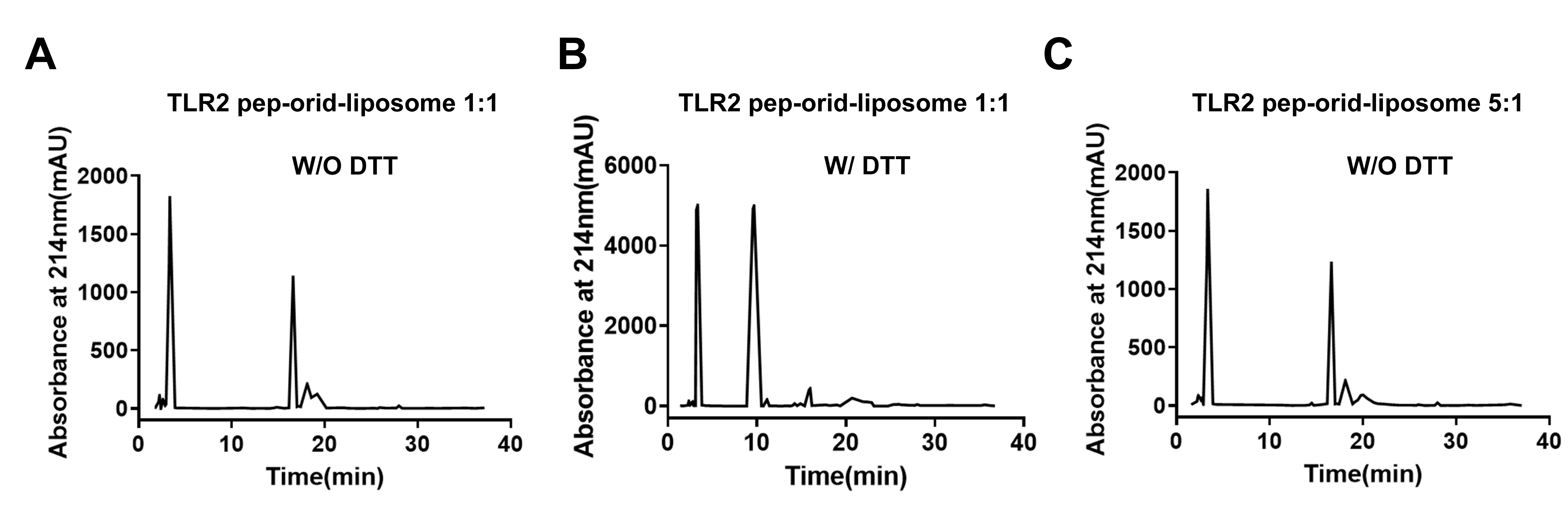
**

**Figure S3.** **Characterization of TLR2 pep-orid-liposome 1:1 and TLR2 pep-orid-liposome 5:1.** TLR2 pep-orid-liposome 1:1 without DTT (A), with 50 mg/ml DTT (B) and TLR2 pep-orid-liposome 5:1 without DTT (C) analyzed by HPLC with detection UV absorbance at 214 nm. Analytical HPLC was performed on a reversed-phase C18 column (Waters XBridge™ 3.5 μm, 4.6 × 150 mm) at 40 °C.

**Figure S4.**

**
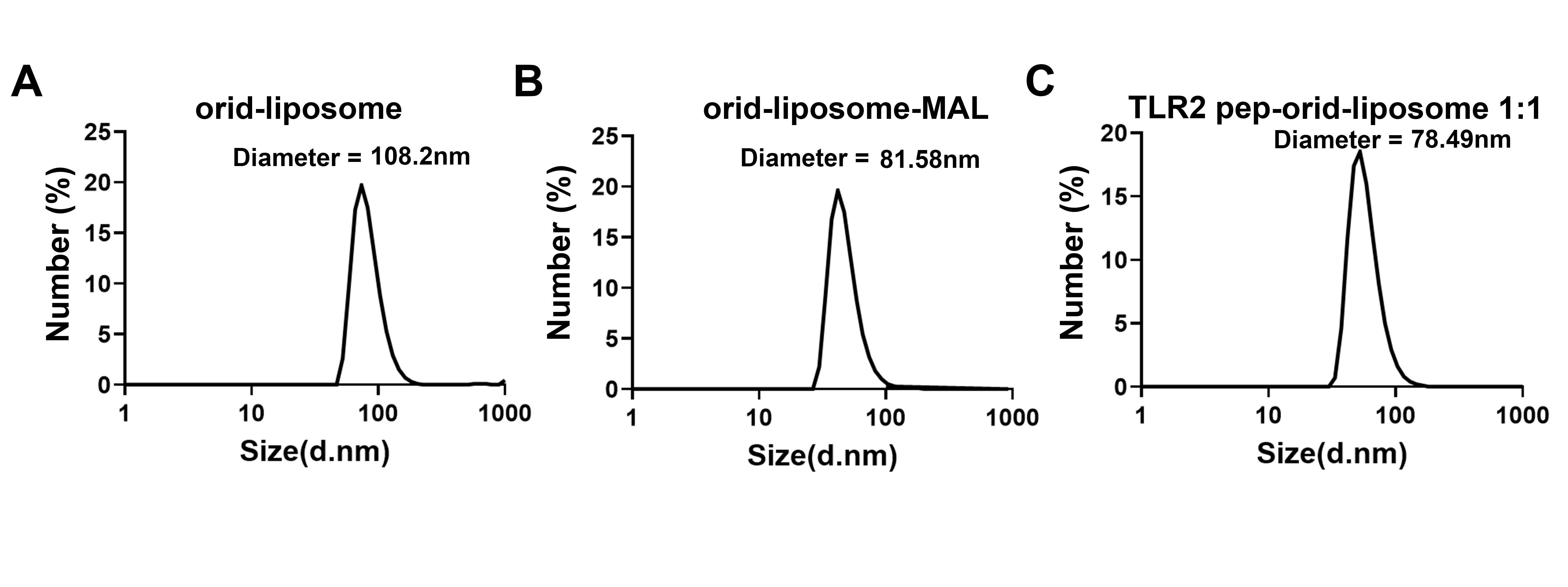
**

**Figure S4. Hydrodynamic diameter of orid-liposome (A), orid-liposome-MAL (B) and TLR2 pep-orid-liposome 1:1 (C)** measured in PBS buffer, pH 7.4, at 25°C by dynamic light scattering.

**Figure S5.**

**
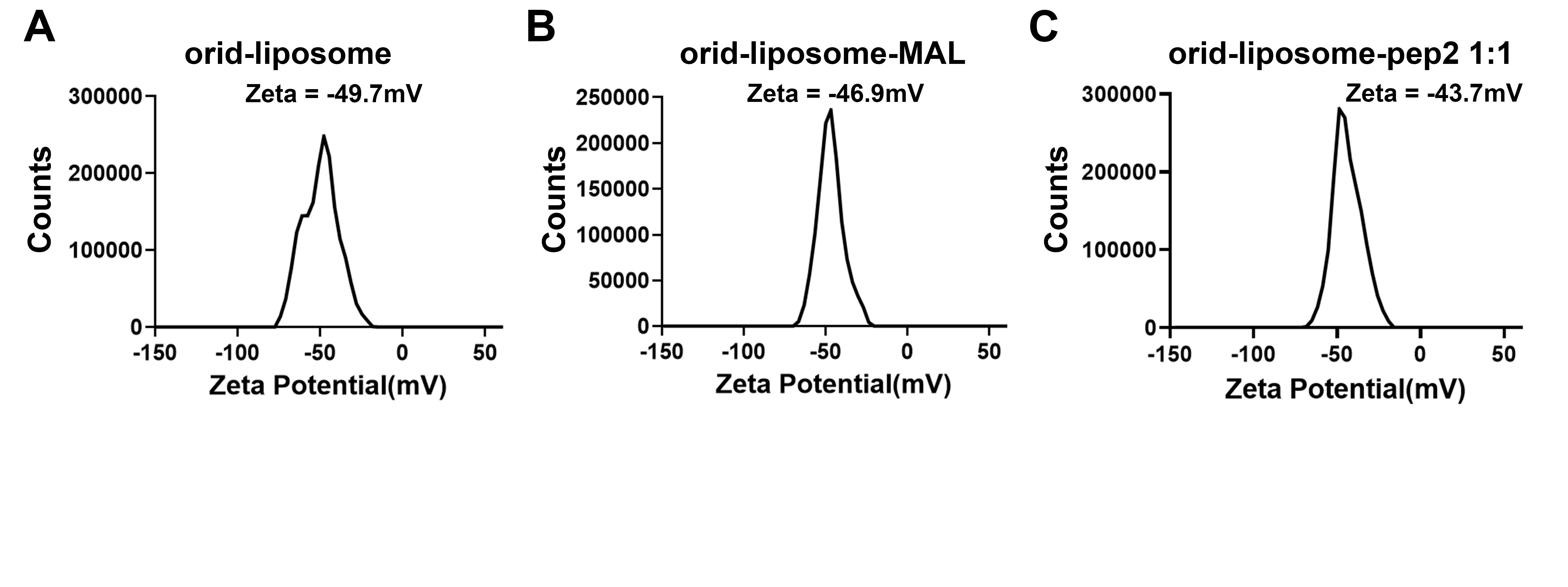
**

**Figure S5. Zeta potential measurement of orid-liposome (A), orid-liposome-MAL (B) and TLR2 pep-orid-liposome 1:1 (C).**

**Figure S6.**

**
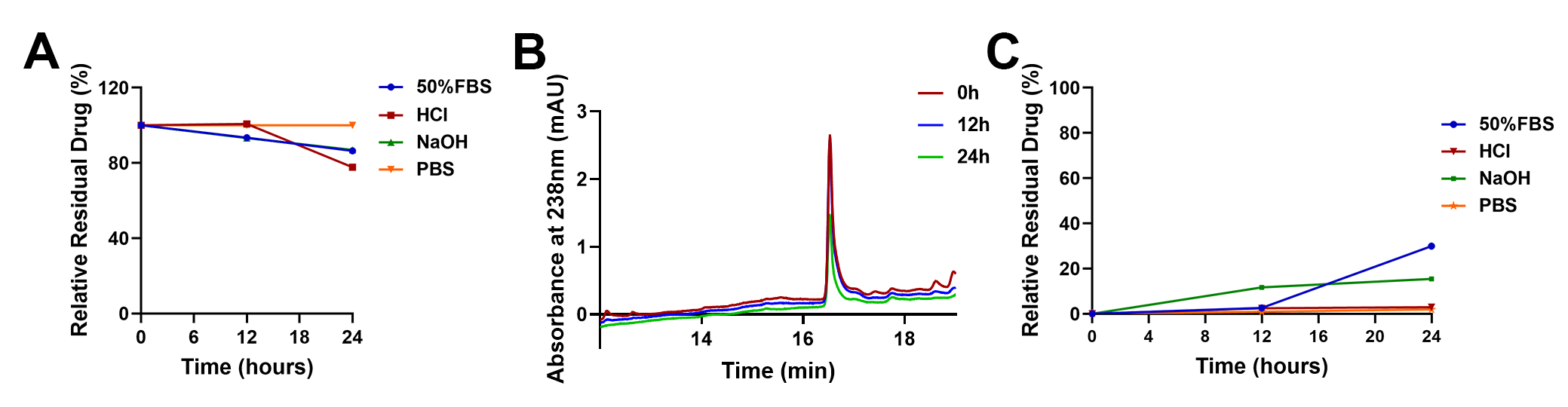
**

**Figure S6. Stability detection and release profiles of TLR2 pep-orid-liposome.** (A) The stability of TLR2 pep-orid-liposomes under different conditions, including exposure to 50% FBS, HCl (pH=3.0), NaOH (pH=10.0), and PBS (pH=7.0). (B) HPLC analysis of residual TLR2 pep-orid-liposome after incubating with 50% FBS. (C) Statistics of HPLC analysis for drug release into the supernatant of TLR2 pep-orid-liposome.

**Figure S7.**

**
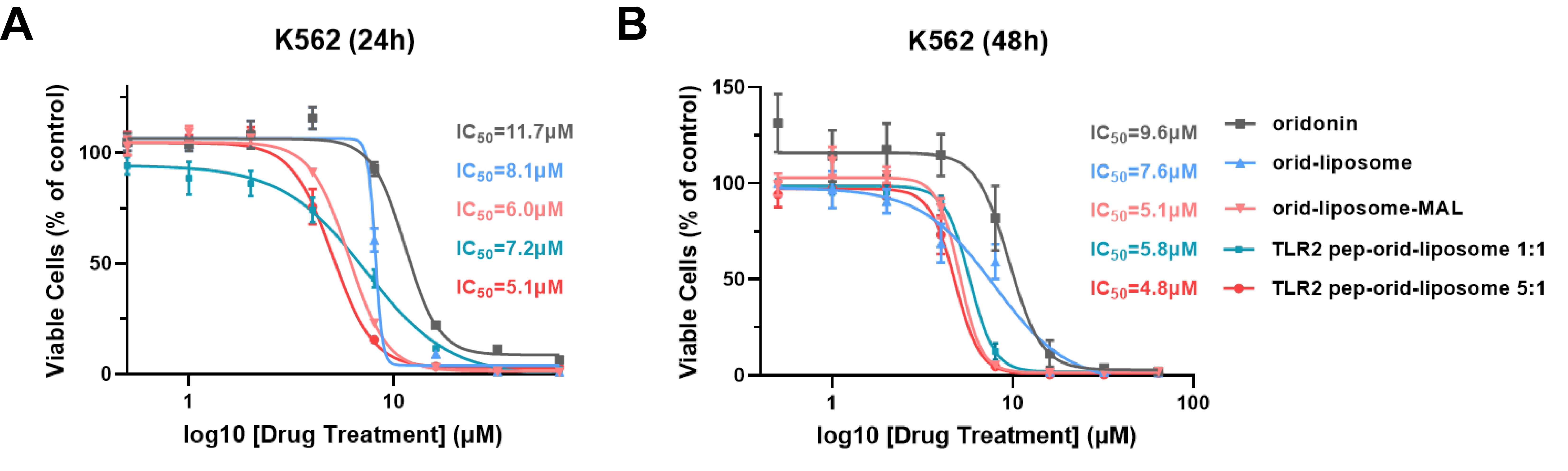
**

**Figure S7. Cell viability test of oridonin, orid-liposome, orid-liposome-MAL, TLR2 pep-orid-liposome 1:1 and TLR2 pep-orid-liposome 5:1 in K562 cell line for treatment 24 hours (A) or 48 hours (B).** (Mean ± SD, n=3).

**Figure S8.**

**
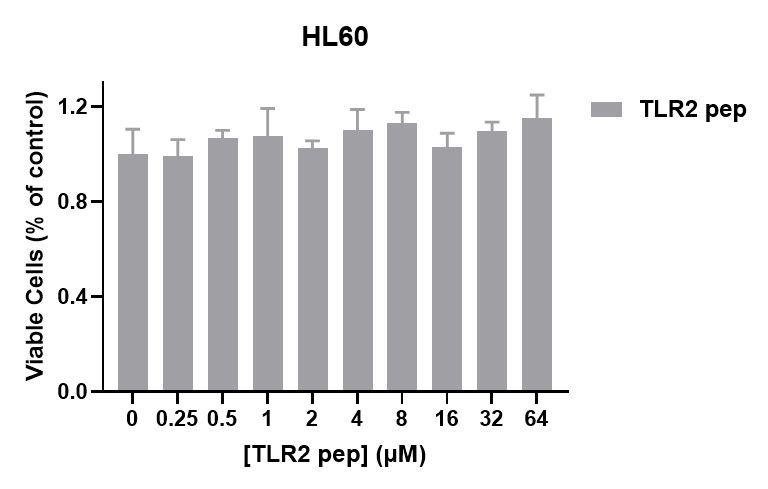
**

**Figure S8. Cell viability test of TLR2 pep-treated HL60 cells for 72 hours.** (Mean ± SD, n=3)**.**

**Figure S9.**

**
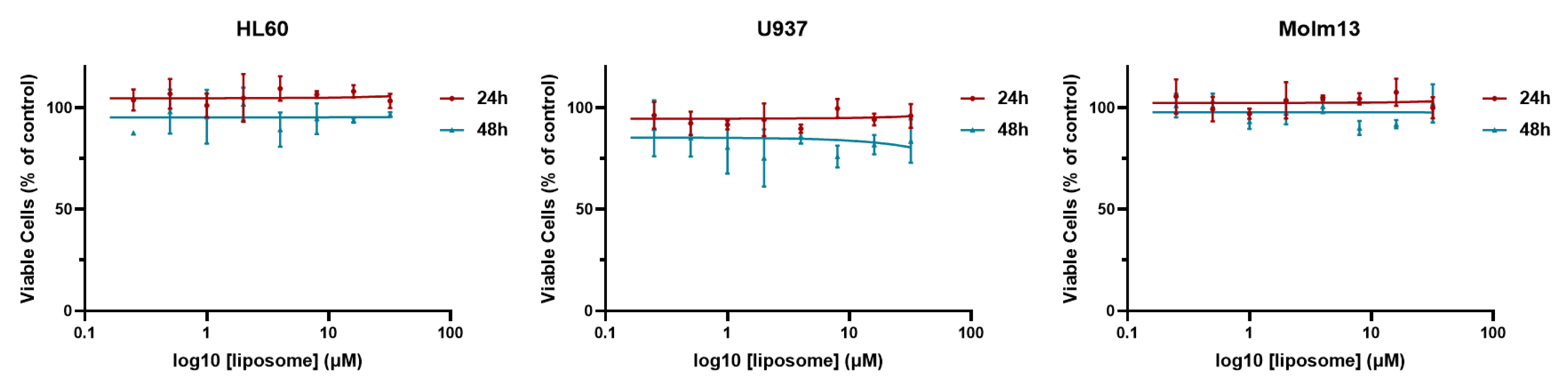
**

**Figure S9. Cell viability test of liposome with non-loaded drugs treated AML cells for 24 hours and 48 hours.** (Mean ± SD, n=3).

**Figure S10.**

**
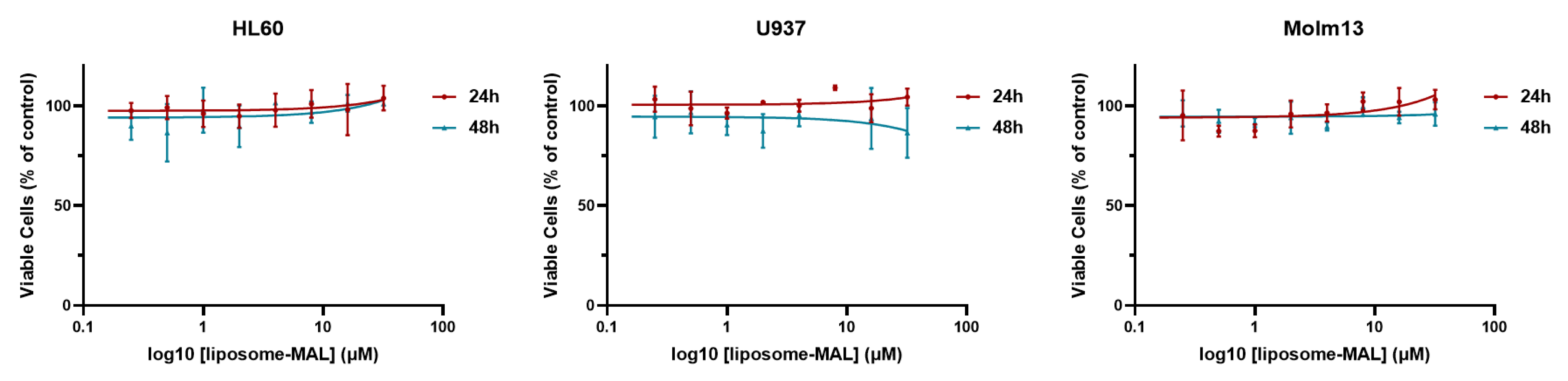
**

**Figure S10. Cell viability test of liposome-MAL with non-loaded drugs treated AML cells for 24 hours and 48 hours.** (Mean ± SD, n=3)**.**

**Figure S11.**

**
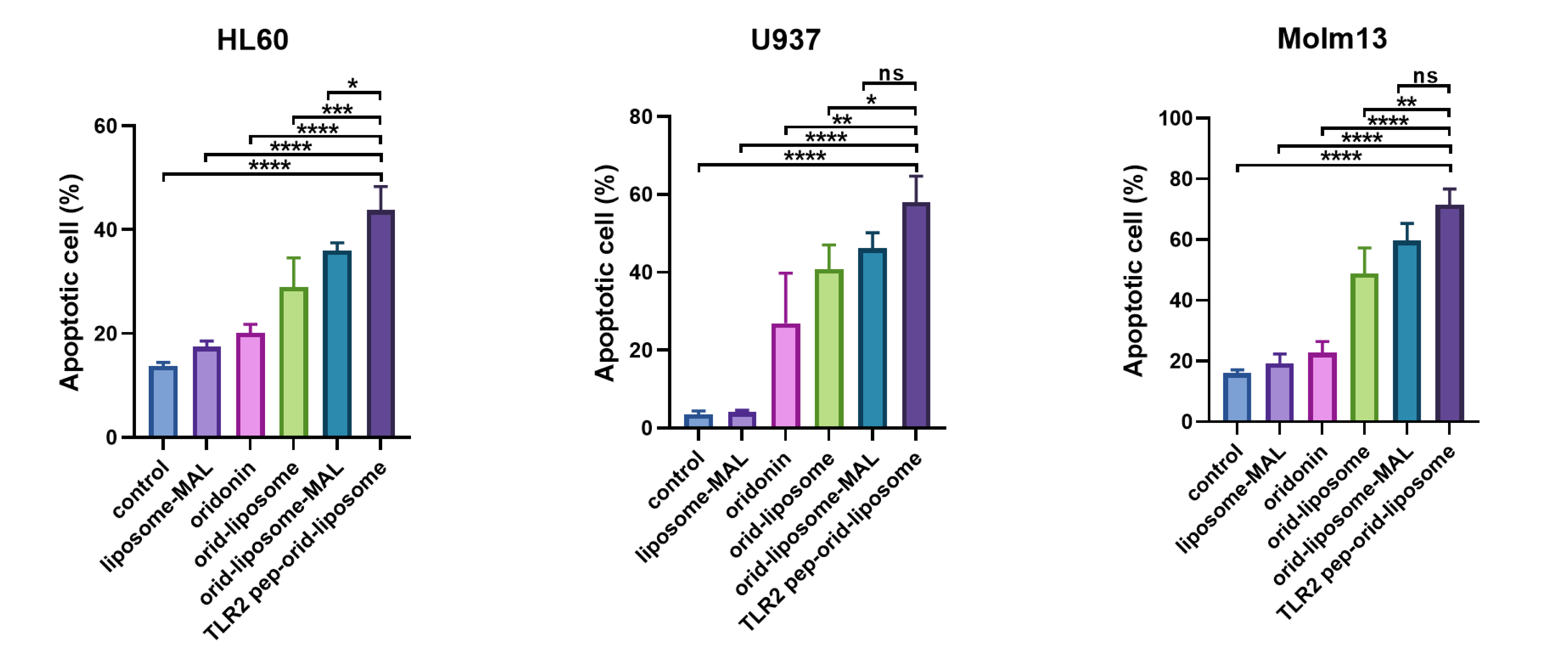
**

**Figure S11.** **Apoptotic analysis and statistical results on apoptosis of HL60, U937 and Molm13 after treatment for 24 hours with liposome-MAL, oridonin, orid-liposome, orid-liposome-MAL or TLR2 pep-orid-liposome (4 μM) by flow cytometry.** (Mean ± SD, n=3; * indicates *p* < 0.05, ** indicates *p* < 0.01, *** indicates *p* < 0.001, **** indicates *p* < 0.0001, ns stands for not statistically significant).

**Figure S12.**

**
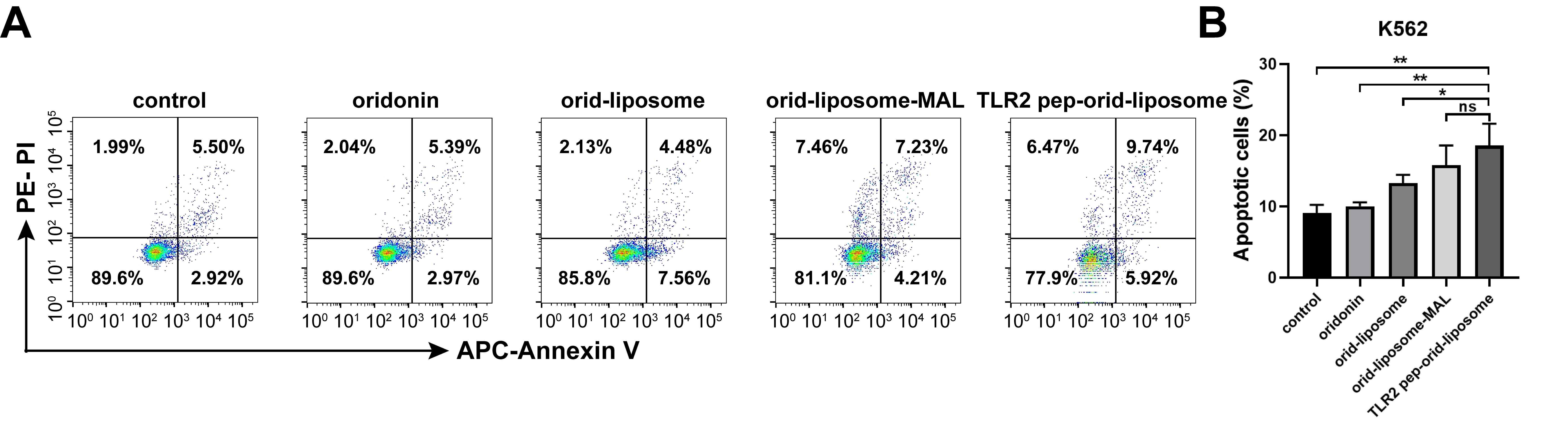
**

**Figure S12.** **Apoptotic analysis and statistical results on apoptosis of K562, 24 hours after treatment with oridonin, orid-liposome, orid-liposome-MAL or TLR2 pep-orid-liposome (4 μM) by flow cytometry.** (Mean ± SD, n=3; * indicates *p* < 0.05, ** indicates *p* < 0.01, ns stands for not statistically significant).

**Figure S13.**

**
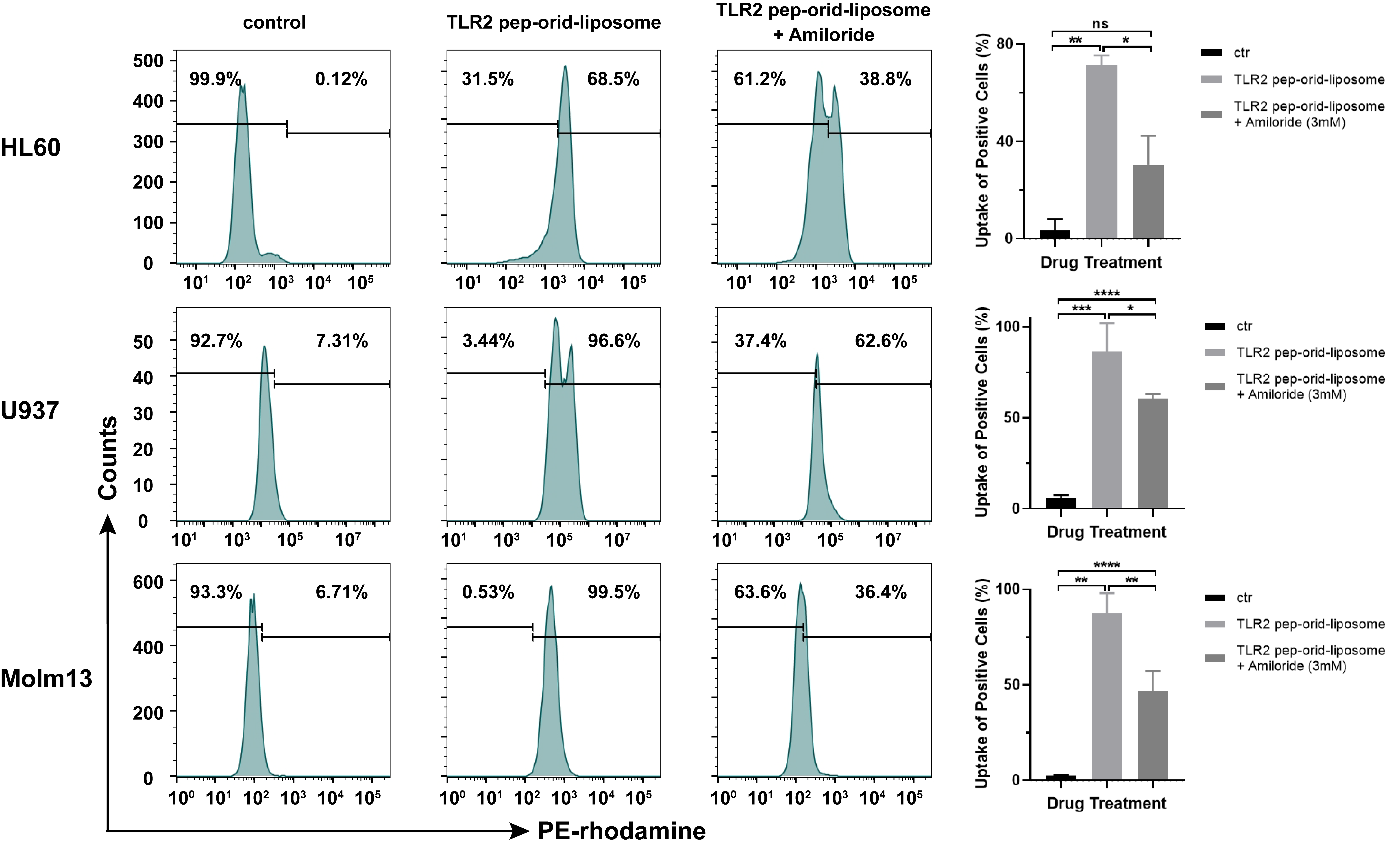
**

**Figure S13. The endocytosis efficiency of rhodamine-labeled TLR2 pep-orid-liposome (4 μM incubated for 2 hours) in different AML cell lines (HL60, U937 and Molm13) was determined by flow cytometry, and endocytosis was inhibited with the endocytotic inhibitor amiloride (3 mM incubated for 12 hours before).** (Mean ± SD, n=3; * indicates *p* < 0.05, ** indicates *p* < 0.01, *** indicates *p* < 0.001, **** indicates *p* < 0.0001, ns stands for not statistically significant).

**Figure S14.**

**
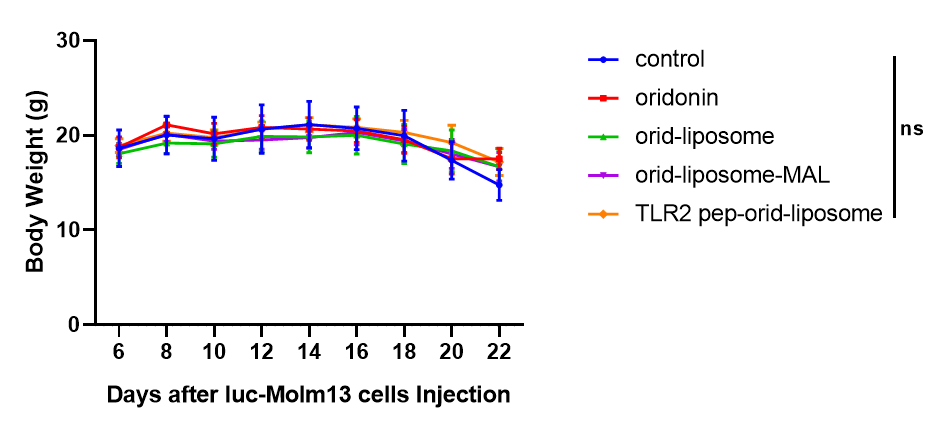
**

**Figure S14. The change of body weight in AML xenograft mouse model during different drug treatments.** (Mean ± SD, n=5; ns stands for not statistically significant).

**Figure S15.**

**
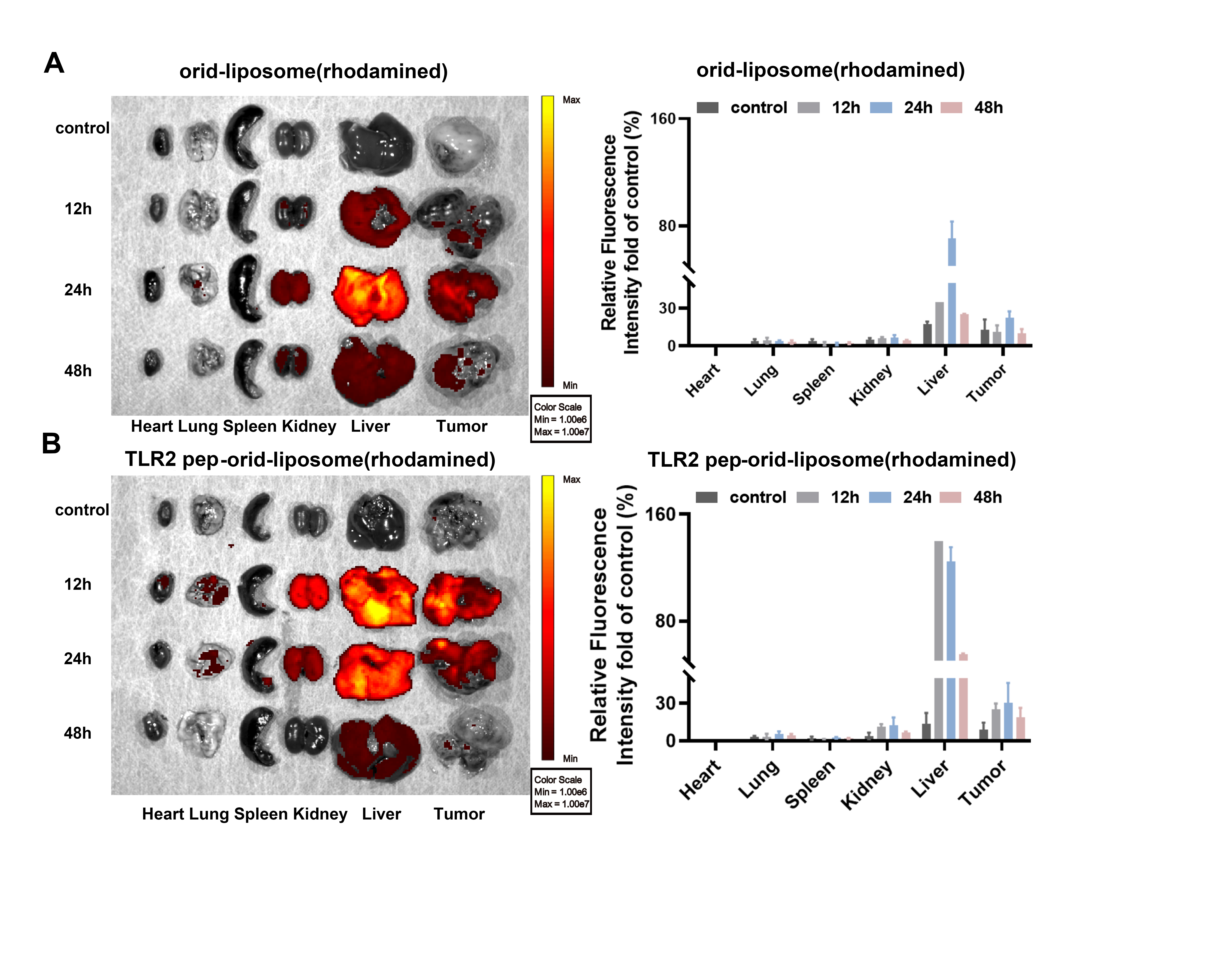
**

**Figure S15. Representative *in vivo* fluorescence images and semi-quantitative analysis of major organs and tumors 12, 24 and 48 hours after intraperitoneal injection of rhodamine-labeled (A) orid-liposome or (B) TLR2 pep-orid-liposome.** Fluorescence intensity in each organ was determined using Living Image 3.0 Software from IVIS fluorescence data expressed as radiant efficiency. (Mean ± SD, n = 3; * indicates *p* < 0.05, ** indicates *p* < 0.01, *** indicates *p* < 0.001, **** indicates *p* < 0.0001).

**Figure S16.**

**
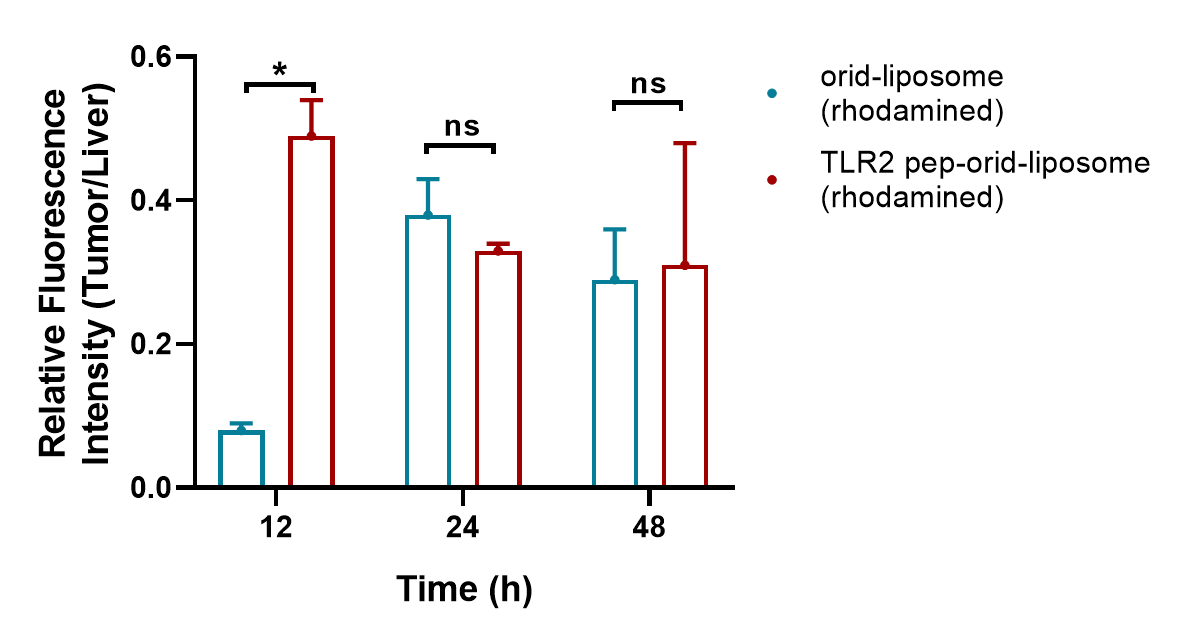
**

**Figure S16. The tumor/liver ratio of the total fluorescence intensity at different time point.** (Mean ± SD, n=3; * indicates *p* < 0.05, ns stands for not statistically significant).

**Figure S17.**

**
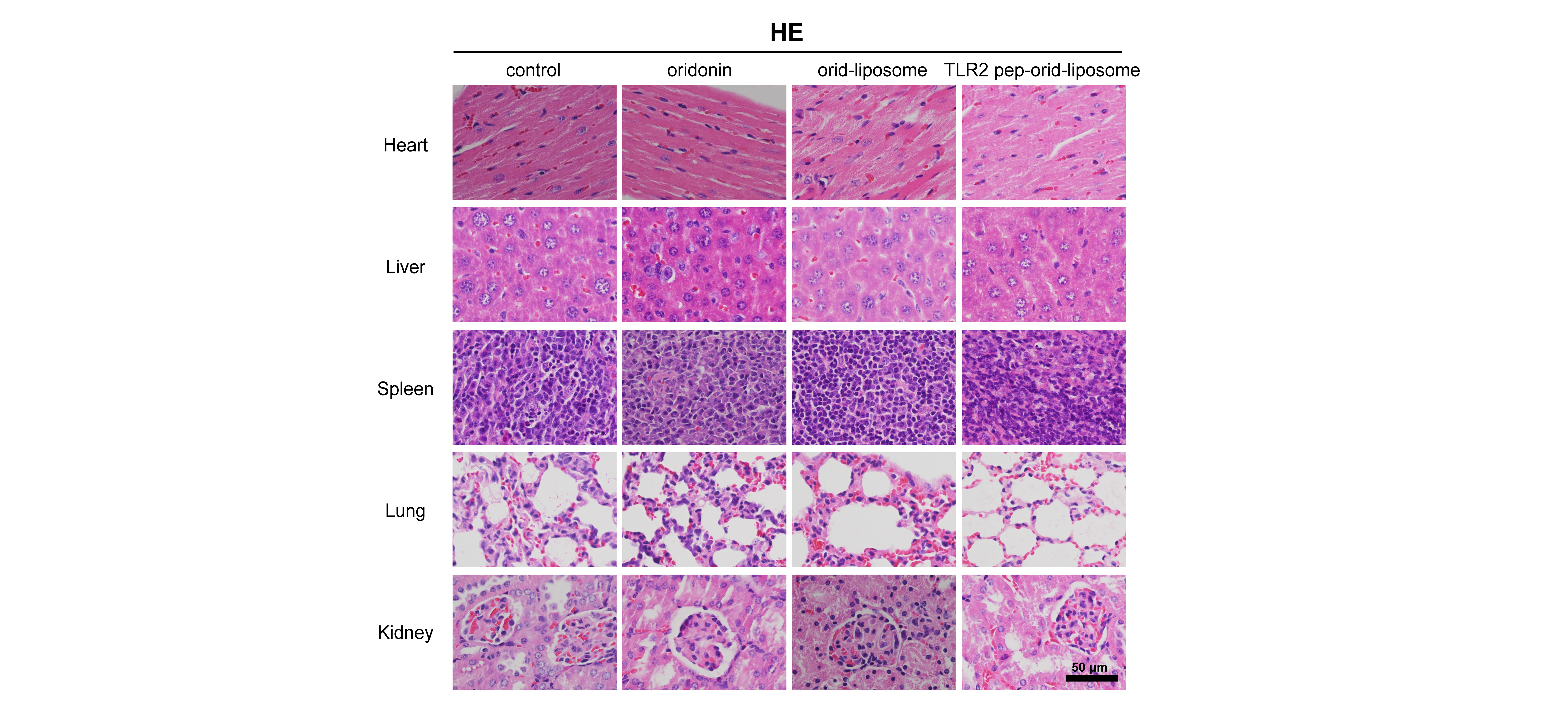
**

**Figure S17. The results of HE staining of various organs to estimate the toxicity of drugs from AML xenograft mouse model.**

**Figure S18.**

**
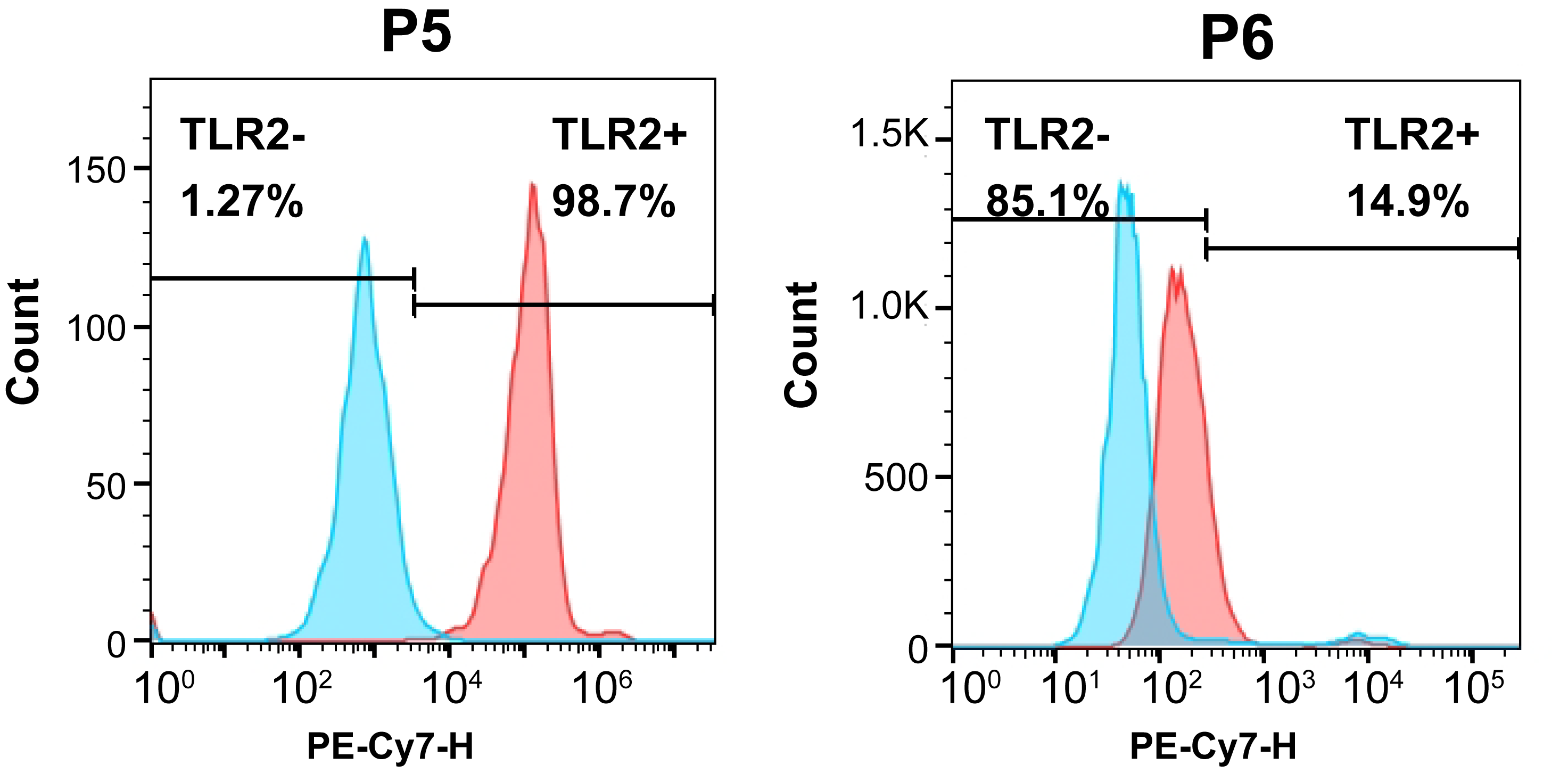
**

**Figure S18. The expression of TLR2 in primary AML cells from patient samples was detected by flow cytometry.**

**Figure S19.**

**
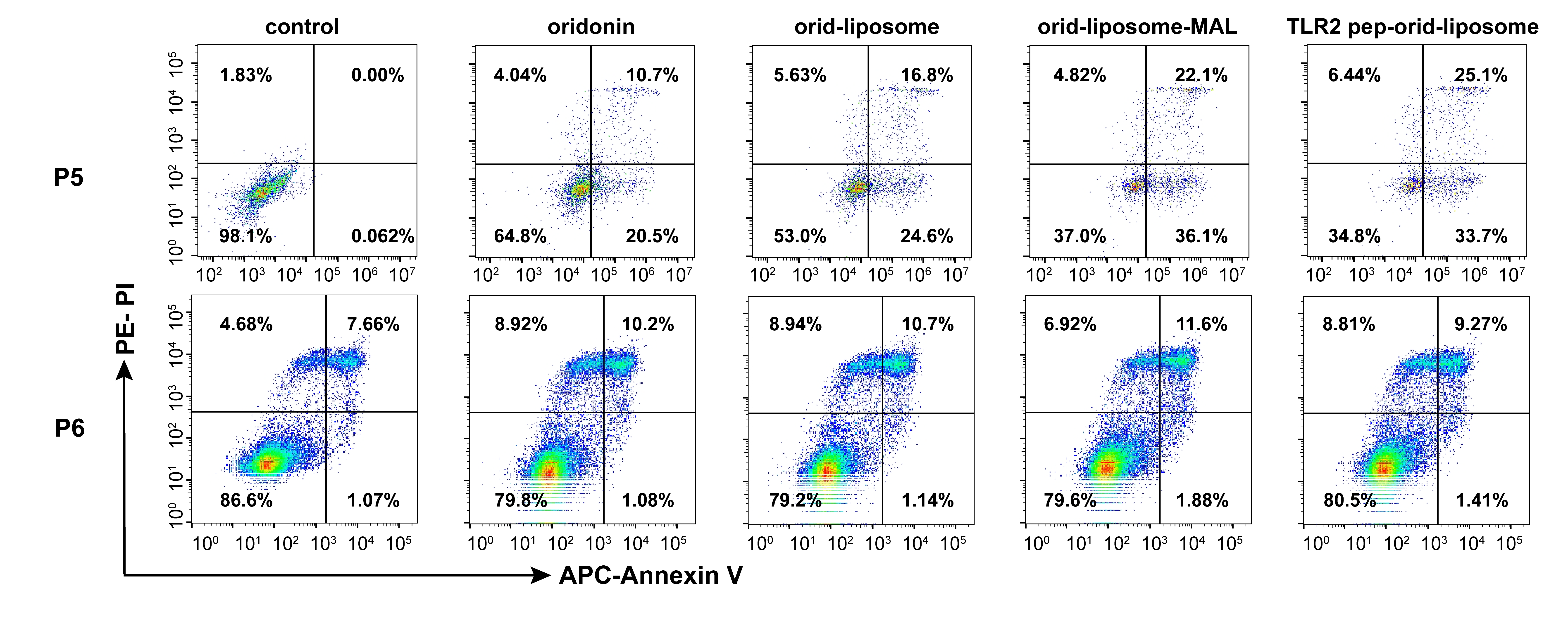
**

**Figure S19. Apoptosis of AML specimens after oridonin, orid-liposome, orid-liposome-MAL or TLR2 pep-orid-liposome treatment was detected by flow cytometry.**

**Figure S20.**

**
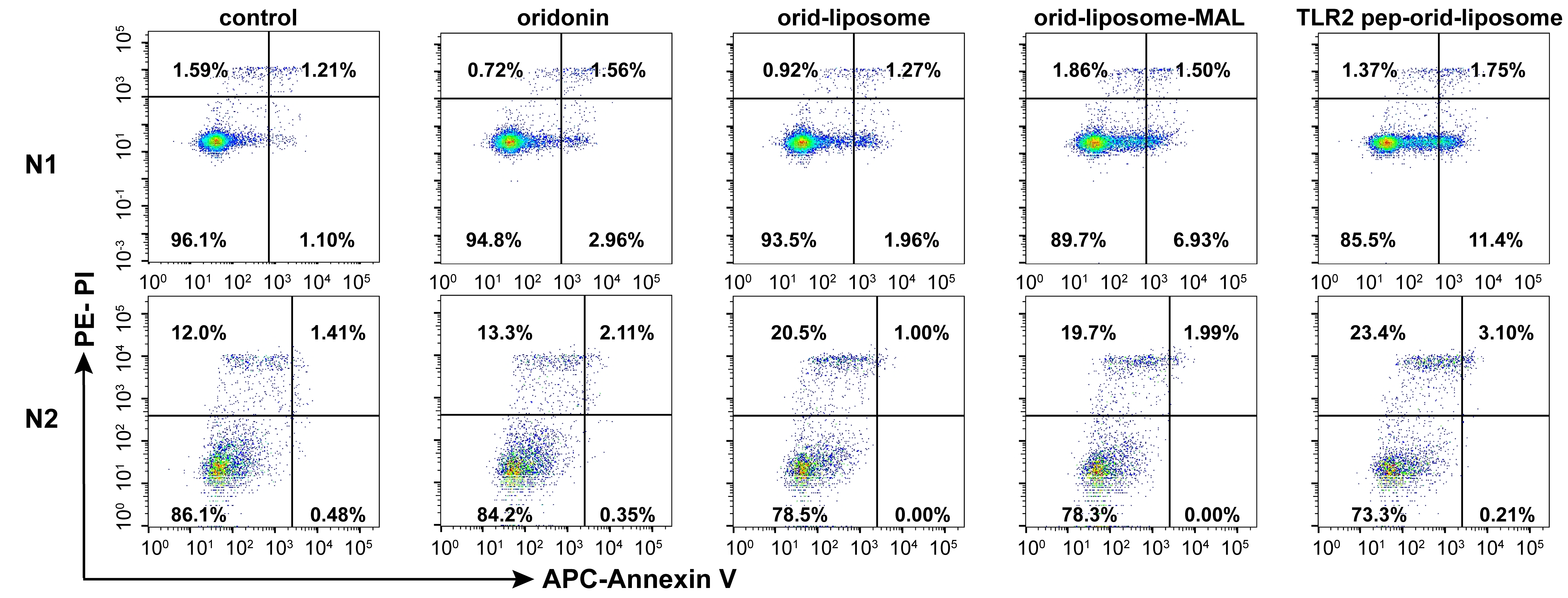
**

**Figure S20.** **Apoptotic analysis of PBMCs from healthy donors after 24-hour treatment with oridonin, orid-liposome, orid-liposome-MAL, or TLR2 pep-orid-liposome (4 μM) by flow cytometry.**

**Table S1. Cell viability tests in HL60, U937, Molm13 and K562 cells of variants form of oridonin (Mean ± SD, n = 3).**

| **Cells** | **Time** | **oridonin**  **(μM)** | **orid-liposome**  **(μM)** | **orid-liposome**  **-MAL (μM)** | **TLR2 pep-**  **orid-liposome**  **1:1 (μM)** | **TLR2 pep-**  **orid-liposome**  **5:1 (μM)** |
| --- | --- | --- | --- | --- | --- | --- |
| **HL60** | **24h** | 6.2 ± 1.5 | 5.9 ± 0.1 | 4.4 ± 0.6 | 2.6 ± 0.2 | 2.6 ± 0.6 |
|  | **48h** | 4.4 ± 0.5 | 3.2 ± 0.1 | 3.5 ± 0.1 | 3.6 ± 0.3 | 2.1 ± 0.1 |
| **U937** | **24h** | 6.7 ± 0.9 | 6.7 ± 0.1 | 5.8 ± 0.7 | 4.1 ± 0.8 | 3.6 ± 0.1 |
|  | **48h** | 4.3 ± 0.7 | 4.2 ± 0.4 | 3.6 ± 0.1 | 4.3 ± 0.4 | 3.7 ± 0.1 |
| **Molm13** | **24h** | 3.9 ± 0.3 | 3.8 ± 0.6 | 3.4 ± 0.6 | 2.9 ± 1.0 | 2.7 ± 0.1 |
|  | **48h** | 3.6 ± 0.9 | 3.2 ± 0.5 | 2.7 ± 0.1 | 2.9 ± 0.3 | 2.5 ± 0.3 |
| **K562** | **24h** | 11.7±0.1 | 7.2±1.5 | 5.6±0.7 | 7.3±0.1 | 4.8±0.3 |
|  | **48h** | 9.4±0.4 | 7.0±0.8 | 4. 9±0.3 | 5.5±0.4 | 4.7±0.1 |

**Table S2. Analysis of combination index (CI) data for non-constant combo: oridonin + liposome-MAL in GSH detection at molecular level (Mean, n = 3).**

| **Dose oridonin (μM)** | **Dose liposome-MAL (mM)** | **Effect** | **CI** |
| --- | --- | --- | --- |
| 15.625 | 1.0 | 0.99044 | 0.06263 |
| 31.25 | 1.0 | 0.99000 | 0.12032 |
| 62.5 | 1.0 | 0.95697 | 0.07672 |
| 125.0 | 1.0 | 0.95697 | 0.15313 |
| 250.0 | 1.0 | 0.88287 | 0.13383 |
| 500.0 | 1.0 | 0.78009 | 0.15036 |
| 1000.0 | 1.0 | 0.58886 | 0.15030 |
| 2000.0 | 1.0 | 0.45500 | 0.19893 |
| 4000.0 | 1.0 | 0.44544 | 0.38624 |

**Table S3**. Basic information of AML patients.

| **Patients** | **Gender** | **Age** | **Diagnosis** | **BM Blasts** | **Immuno-phenotyping** | **Fusion Genes** | **Mutations** | **Chromosomal Abnormalities** | **TLR2**  **expression** |
| --- | --- | --- | --- | --- | --- | --- | --- | --- | --- |
| **P1** | Male | 14 | AML-M5b | 98.5% | HLA-DR、CD4、CD11b、CD15、CD33、CD38、CD64、CD123、CD56(+) | (-) | MLL-AF9(+) | (-) | High |
| **P2** | Female | 52 | AML-M1 | 88% | HLA-DR, CD33, CD34, CD38, CD117, CD123 (+) | MLL-AF6/WT-1（+） | (-) | (-) | High |
| **P3** | Male | 36 | AML-M3 | 85% | CD9, CD13, CD33, CD38, CD64, CD117, CD123, MPO (+) | (-) | (-) | (-) | Low |
| **P4** | Male | 28 | AML-M5 | 93% | HLA-DR, CD4, CD33, CD38, CD64,  CD123 (+) | (-) | FLT3-ITD3(+) | (-) | Low |
| **P5** | Female | 38 | MDS-AML  Transform | 84% | HLA-DR, CD7, CD13, CD33, CD34, CD38, CD117, CD123, MPO (+) | WT-1(+) | CEBPA/STAG2(+) | (-) | High |
| **P6** | Female | 62 | AML-M5b | 96.5% | HLA-DR、CD4、CD11b、CD13、CD14、  CD15、CD33、  CD38、CD64、CD123(+) | Undetected | Undetected | Undetected | Low |

**Table S4. Basic information of PBMCs from healthy donors.**

| **Name** | **Gender** | **Age** | **Cell Type** | **Cell origin** |
| --- | --- | --- | --- | --- |
| **N1** | Female | 28 | Peripheral Blood Mononuclear Cell (PBMC) | Healthy Donor |
| **N2** | Male | 31 | Peripheral Blood Mononuclear Cell (PBMC) | Healthy Donor |
